# Sex in Symbiodiniaceae dinoflagellates: genomic evidence for independent loss of the canonical synaptonemal complex

**DOI:** 10.1038/s41598-020-66429-4

**Published:** 2020-06-17

**Authors:** Sarah Shah, Yibi Chen, Debashish Bhattacharya, Cheong Xin Chan

**Affiliations:** 1 Institute for Molecular Bioscience, The University of Queensland, Brisbane, QLD 4072 Australia; 2 School of Chemistry and Molecular Biosciences, The University of Queensland, Brisbane, QLD 4072 Australia; 3 Australian Centre for Ecogenomics, The University of Queensland, Brisbane, QLD 4072 Australia; 4 Department of Biochemistry and Microbiology, Rutgers University, New Brunswick, NJ 08901 USA

**Keywords:** Genomics, Evolutionary genetics, Microbial genetics, Comparative genomics

## Abstract

Dinoflagellates of the Symbiodiniaceae family encompass diverse symbionts that are critical to corals and other species living in coral reefs. It is well known that sexual reproduction enhances adaptive evolution in changing environments. Although genes related to meiotic functions were reported in Symbiodiniaceae, cytological evidence of meiosis and fertilisation are however yet to be observed in these taxa. Using transcriptome and genome data from 21 Symbiodiniaceae isolates, we studied genes that encode proteins associated with distinct stages of meiosis and syngamy. We report the absence of genes that encode main components of the synaptonemal complex (SC), a protein structure that mediates homologous chromosomal pairing and class I crossovers. This result suggests an independent loss of canonical SCs in the alveolates, that also includes the SC-lacking ciliates. We hypothesise that this loss was due in part to permanently condensed chromosomes and repeat-rich sequences in Symbiodiniaceae (and other dinoflagellates) which favoured the SC-independent class II crossover pathway. Our results reveal novel insights into evolution of the meiotic molecular machinery in the ecologically important Symbiodiniaceae and in other eukaryotes.

## Introduction

Sex is part of the life cycle of nearly all eukaryotes and has most likely been so since the last eukaryotic common ancestor^1^. Even lineages that were traditionally thought to be asexual, such as the Amoebozoa, possess the molecular machinery required for sex^2^. Dinoflagellates, a group of flagellated, mostly marine phytoplankton, are no exception. From the deeper-branching species *Gymnodinium catenatum* to the more recently-diverging *Alexandrium minutum*, syngamy and meiosis have been observed cytologically and through mating-type experiments^3^. The family Symbiodiniaceae, a lineage that branches between these two^4^, has been suggested to be sexual based on an early life-cycle description^5^. Some species of Symbiodiniaceae are symbionts, associated with a wide range of coral reef organisms, including cnidarians, molluscs, and foraminifera. Importantly, the dissociation of Symbiodiniaceae from reef-building corals under environmental stress (i.e., coral bleaching) can lead to coral death and eventual collapse of coral reefs^6^. A thorough understanding of the molecular mechanisms that underpin the reproduction of Symbiodiniaceae will elucidate the selective forces acting on this trait and adaptation of these ecologically important taxa.

Incongruence between the phylogenies of multiple isoenzymes and the internal transcribed spacer (ITS) regions^7^, and between the phylogenies of organellar and nuclear gene markers^8^ suggests that Symbiodiniaceae undergo hybridisation, in addition to clonal propagation. The identification of many meiotic toolkit genes in four diverse Symbiodiniaceae species (*Symbiodinium microadriaticum*, *Breviolum minutum*, *Cladocopium goreaui*, and *Fugacium kawagutii*) supports the notion that Symbiodiniaceae may be sexual^9,10^. Symbiodiniaceae cells grow as motile, flagellated cells (mastigotes) under light and divide in the dark as coccoid cells^11^. Symbiodiniaceae are believed to be isogamous^5^, but it is difficult to confirm through direct observation if these cell divisions are mitotic or meiotic. If sex occurs during the dark part of the life cycle as found in *A. minutum*^3^, one may assume that dinoflagellates including Symbiodiniaceae can respond to selection pressure by producing genetic variation through sexual recombination^12^. Some genes have a preferred set of codons due to variable abundance of distinct tRNAs; this codon usage bias affects the efficiency of gene expression. In mammals, codon usage bias was hypothesised to be a result of GC-biased gene conversion driven by meiotic recombination^13^. A higher rate of recombination does not necessarily improve the efficacy of selection, or of the removal of deleterious mutations^14^. Nevertheless, a strong codon usage bias likely arose via positive selection, and would inform us about processes that have been favoured in the evolution of Symbiodiniaceae.

One of the hallmarks of meiosis is the formation of the synaptonemal complex (SC), a proteinaceous scaffolding that juxtaposes homologous chromosomes, mediating their synapsis (i.e., the pairing of chromosomes for potential crossovers) during prophase I. The conventional SC consists of tripartite proteinaceous elements in parallel: a central element and two outer lateral elements, like two ladders attached side-to-side, with rungs of transversal filament proteins holding the elements together. Loops of sister chromatids are tethered to each lateral element. The process of synapsis “zips” down the scaffolding, with crossovers occurring at recombination nodules. Unsynapsed regions of the lateral elements are referred to as axial elements (see Loidl^15^ for a detailed diagram). These axial elements comprise **ho**mologous **p**airing protein **1** (Hop1) and **red**uctional division protein **1** (Red1), whereas **p**achytene **ch**eckpoint protein **2** (Pch2) prevents chromosome segregation when synapsis and recombination are defective^16^. Formation of the SC is triggered by the synapsis initiation complex proteins, more commonly known as the ZMM proteins: (a) the transversal filament protein Zip1 (not to be confused with zinc transporter Zip1) attaches the central element to the pair of lateral elements; (b) Zip2, Zip3, and Zip4 mediate protein-protein interaction; (c) Mer3, a DNA helicase unwinds double-stranded DNA; and (d) the Msh4-Msh5 heterodimer binds to Holliday junction^17^. Metazoan equivalents of these proteins are named **sy**naptonemal **c**omplex **p**roteins (SYCP) and **sy**naptonemal **c**omplex central **e**lement (SYCE) proteins. Genes encoding the SC and ZMM proteins are meiosis-specific and have been used as indicators of sex in diverse eukaryotes^2^.

Some genes, although not strictly in meiosis-specific pathways, are also relevant to meiosis. For example, **p**ost**m**eiotic **s**egregation increased homologs **1** and **2** (*PMS1* and *PMS2*) are part of the DNA mismatch repair system during both mitosis and meiosis. They compete to heterodimerise with **M**ut**L h**omolog **1** (*MLH1*), which is then assembled into the MutL-MutS heteroduplex that aids in degradation of DNA strands. Genes involved in syngamy (i.e., gamete fusion during fertilisation) are also relevant to sexual reproduction. Cell membranes fuse first (plasmogamy), followed by nuclear fusion (karyogamy); genes associated with these processes include *HAP2* (**hap**loid-disrupting 2) and **g**amete **ex**pressed protein **1** (*GEX1*). Thought to be an ancestral gene in all eukaryotes, *HAP2* encodes the transmembrane protein Hap2-GCS1 that inserts into the target membrane using a hydrophobic fusion loop^18^. *GEX1* gene products are nuclear envelope proteins involved in karyogamy^19^.

To attain a comprehensive overview of sex and reproduction in Symbiodiniaceae, here we used available genome and transcriptome data from broadly sampled taxa to investigate the presence of genes that encode functions critical to different stages of meiosis and syngamy, and their associated protein complexes. We also investigated codon usage in nuclear genes from each isolate, and compared the predicted functions between genes with strong codon usage preference and those under neutral selection.

## Results

### Canonical synaptonemal complex is absent in Symbiodiniaceae

**Figure 1:**
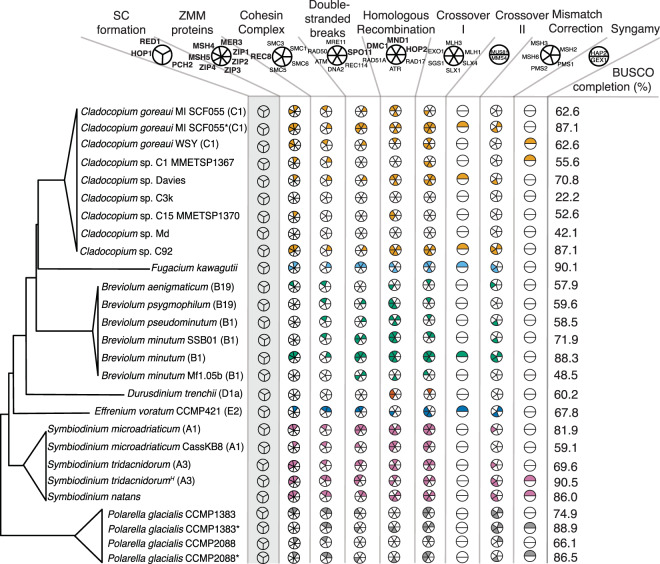
Sex-associated gene inventory of the 21 Symbiodiniaceae isolates analysed in this study, using *Polarella glacialis* isolates as outgroups. For *Cladocopium goreaui* MI SCF055, *Polarella glacialis* CCMP1383, and *Polarella glacialis* CCMP2088, both transcriptomes and predicted proteins were searched, with the latter versions marked with asterisks. *Symbiodinium tridacnidorum*^*H*^ denotes proteins predicted from the hybrid genome assembly; *Symbiodinium tridacnidorum* denotes proteins predicted from the short-read only assembly. BUSCO completion includes both complete and fragmented orthologs. The tree topology shown on the left is based on LaJeunesse *et al*.^69^; branch lengths are not to scale. Distinct processes or protein complexes attributed to meiosis and fertilisation are shown at the top, with pie charts beneath showing genes associated with each process/complex. Genes in boldface are meiosis-specific. Genes under “double-stranded breaks” refer to those involved in inducing double-stranded DNA breaks while those under “homologous recombination” are involved in repairing said breaks. Presence of a gene is represented by coloured sections of a pie chart, with different colours assigned to different genera (e.g., *Cladocopium goreaui* has *MSH5* and *MER3* from the ZMM proteins). Absence of the genes *HOP1*, *RED1*, and *PCH2*, which are essential in the formation of the SC, is shown against a grey background.

The datasets of 21 isolates of Symbiodiniaceae used in this study are shown in Table 1. We found that meiosis-specific genes involved in the formation of the synaptonemal complex (SC) were largely missing from these microalgae. We did not recover genes encoding Hop1, Red1, Pch2, Zip1, Zip2, Zip3, and Zip4 (Fig. 1). However, we identified some of the genes that encode the ZMM proteins: the DNA helicase Mer3 and the Holliday junction heterodimer Msh4-Msh5. We also found most of the representative genes involved in homologous recombination, i.e., *HOP2, MND1, DMC1*, *RAD51A* and *ATR*. This result suggests that Symbiodiniaceae is capable of producing genetically diverse gametes in the absence of a canonical SC. This pattern of non-SC mediated meiotic crossovers may also hold for other dinoflagellates; *Gymnodinium pseudopalustre* Schiller, *Amphidinium cryophilum*, *Alexandrium tamarense* (basionym *Gonyaulax tamarensis*), *Alexandrium minutum*, and several species of Tovelliaceae (basionym *Woloszynska*) have all been observed to lack obvious SC or SC-like structures^3,20–22^. The lack of SC proteins in dinoflagellates was previously described^23^, but the studied taxa remain unspecified, and the genetic resources from dinoflagellates were very limited at that time, with no genome-scale data available. Table 2 shows the loss of canonical SCs reported in eukaryote lineages.

**Table 1:** Dataset used in this study. MMETSP: Marine Microbial Eukaryote Transcriptome Sequencing Project. For *C. goreaui* and *P. glacialis*, asterisks indicate predicted protein versions from genome data of the same isolate, in addition to transcriptome data.

Isolate	Type	GC-content of CDS (%)	Source
*Cladocopium* sp. C1 MMETSP1367	Transcriptome	54.7	MMETSP
*Cladocopium* sp. C15 MMETSP1370	Transcriptome	54.4	MMETSP
*Cladocopium* sp. Davies	Transcriptome	54.9	^70^
*Cladocopium* sp. C3k	Transcriptome	55.0	^71^
*Cladocopium* sp. Md	Transcriptome	53.8	^72^
*Cladocopium goreaui* MI SCF055 (C1); Magnetic Island isolate, same isolate as used in Liu *et al*.^10^	Transcriptome	54.9	^73^
*Cladocopium goreaui* SM (C1); South Molle isolate, also known as Whitsunday Islands (WSY) isolate	Transcriptome	54.5	^73^
*Breviolum aenigmaticum* (B19)	Transcriptome	51.7	^74^
*Breviolum* sp. SSB01 (B1)	Transcriptome	51.6	^75^
*Breviolum* sp. B1 Mf1.05b (B1)	Transcriptome	50.5	^54^
*Breviolum pseudominutum* (B1)	Transcriptome	51.8	^74^
*Breviolum psygmophilum* (B19)	Transcriptome	51.7	^74^
*Durusdinium trenchii* (D1a)	Transcriptome	55.1	MMETSP
*Effrenium voratum* (E2)	Transcriptome	58.6	MMETSP
*Symbiodinium microadriaticum* CassKB8 (A1)	Transcriptome	56.7	^54^
*Polarella glacialis* CCMP1383	Transcriptome	58.1	MMETSP
*Polarella glacialis* CCMP2088	Transcriptome	57.4	MMETSP
*Polarella glacialis* CCMP1383*	Predicted proteins	57.8	^43^
*Polarella glacialis* CCMP2088*	Predicted proteins	57.8	^43^
*Breviolum minutum* (B1)	Predicted proteins	51.2	^26,76^
*Symbiodinium tridacnidorum* (A3)	Predicted proteins from hybrid assembly	57.3	^24^
*Symbiodinium tridacnidorum* (A3)	Predicted proteins	57.8	^26,77^
*Cladocopium* sp. C92	Predicted proteins	54.1	^26,77^
*Symbiodinium microadriaticum* (A1)	Predicted proteins	57.4	^26,61^
Cladocopium goreaui* MI SCF055 (C1)	Predicted proteins	56.4	^10,26^
*Fugacium kawagutii*	Predicted proteins	55.1	^10,26^
*Symbiodinium natans*	Predicted proteins from hybrid assembly	58.2	^24^

**Table 2:** Eukaryotic organisms that lack a synaptonemal complex and the supporting lines of evidence.

	Microscopic observation	Evidence *in silico*
Ciliates	Loidl and Scherthan^78^ observed no SC for *Tetrahymena thermophila*, but Chi *et al*.^40^ observed fragmented SCs in *Stylonychia mytilus*, a close relative of *Oxytricha trifallax*.	*Tetrahymena thermophila*, *Paramecium tetraurelia*, *Ichthyophthirius multifiliis*, and *Oxytricha trifallax*^40^
Dinoflagellates	*Gymnodinium pseudopalustre*, *Amphidinium cryophilum*, *Alexandrium tamarense*, *Alexandrium minutum*, and several species of Tovelliaceae^3,20–22^	In unspecified taxa^23^
Diatoms	Unconfirmed. Frustule and dense chromatin obstruct view of potential SCs^79,80^. Manton *et al*.^81^ observed SC-like ribbons in *Lithodesmium undulatum*.	*Pseudo-nitzschia multistriata, Seminavis robusta*, *Fragilariopsis cylindrus*, *Thalassiosira pseudonana*, and *Phaeodactylum tricornutum*^48^
Corn smut fungus *Ustilago maydis*	^49^	^50,82^
Fission yeast *Schizosaccharomyces pombe*	^45,83^	^45^
Filamentous fungus *Aspergillus nidulans*	^84^	Putative genes encoding Hop1, Red1, and Mer3 (GenBank: CBF81757.1, EAA61648.1, CBF81763.1) are present. Genes encoding Pch2, Zip1, Zip2, Zip3, and Zip4 are absent.
Male fruit fly *Drosophila melanogaster*	^85^	Gilboa and Lehmann^86^ showed molecular pathways taken during spermatogenesis versus oogenesis.

### Genes playing major roles in meiosis and syngamy are present

We identified the gamete fusogen gene, *HAP2*, in the transcriptomes of *Cladocopium* sp. SM (also known as isolate WSY) and *Cladocopium* sp. C1 MMETSP1367, and in the genomes of *Symbiodinium natans* and *Symbiodinium tridacnidorum* (both high-quality hybrid-reads assemblies^24^). This suggests that some of the cells observed to be attached together, i.e., “large tetrads” described in early microscopic observations may indeed be fertilisation. It is unsurprising that *HAP2* occurs in Symbiodiniaceae, as this gene has been observed in other taxa in the Alveolata (to which dinoflagellates also belong): the ciliate *Tetrahymena thermophila* (UniProtKB: HAP2_TETTH, “evidence at protein level”) and in the apicomplexan *Plasmodium berghei* (UniProtKB: HAP2_PLABA, “evidence at transcript level”). We also recovered *HAP2* candidates in other dinoflagellates, namely *Polarella glacialis* CCMP1383, *Prorocentrum minutum*, *Gymnodinium catenatum*, *Noctiluca scintillans*, and *Oxyrrhis marina*; its presence in the latter two species has been independently confirmed by Hofstatter and Lahr^25^. See Supplementary Fig. S1 online for a phylogeny showing the clustering of alveolate Hap2 sequences. Although we did not find clear evidence of *HAP2* among the predicted gene models from all six available symbiodiniacean genomes^26^, we recovered fragments of this gene in the transcriptomes of *S. natans* and *S. tridacnidorum*. The presence of *HAP2* remains to be more thoroughly investigated as more high-quality genomes become available to guide gene prediction methods.

We did not recover the karyogamy gene *GEX1* in all the isolates studied here, but its absence does not necessarily mean Symbiodiniaceae do not undergo nuclear fusion. The malaria-causing parasite *Plasmodium falciparum*, the ciliate *Tetrahymena thermophila*, the diatom *Thalassiosira pseudonana*, and the blight-causing oomycete *Phytophthora infestans* also appear to be missing *GEX1*, but gamete fusion is well-established in these cases.

Symbiodiniaceae appear to have a reduced set of cohesin complex genes. We found only genes encoding the main component of the cohesin complex, the heterodimer Smc1-Smc3, which forms a proteinaceous ring around sister chromosomes or sister chromatids. We did not find *REC8*, *SMC5*, or *SMC6*. Because *REC8* plays a role in anaphase I (separating homologous chromosomes) and anaphase II (separating sister chromatids), this raises the possibility that Symbiodiniaceae may not go through the same mechanism of chromosome/chromatid separation in canonical meiosis. In canonical meiosis, the Smc5-Smc6 heterodimer (encoded by *SMC5* and *SMC6*) recruits Smc1-Smc3 to double-strand DNA breaks. It appears that in Symbiodiniaceae, Smc5-Smc6 has been replaced by another protein complex, or the recruitment process does not occur.

We found candidate genes for the mismatch repair proteins Pms1 and Pms2 in Symbiodiniaceae; these sequences were clustered as a single family (see Supplementary Fig. S2 online). In comparison, the opisthokont counterparts are sufficiently distinct as separate subfamilies (see PANTHER family tree PTHR10073). Searching against the KEGG ortholog HMM database (KOfam), these candidate proteins in Symbiodiniaceae shared higher sequence similarity to “DNA mismatch repair protein PMS2” (KOfam ID: K10858) than to “DNA mismatch repair protein PMS1” (KOfam ID: K10864); we thus annotated them as Pms2 here. Since Pms1 and Pms2 play the same role in DNA mismatch repair, it is unlikely that the absence of either one dramatically affects meiosis in Symbiodiniaceae.

Other genes that we did not recover in Symbiodiniaceae or *P. glacialis* are *DNA2* and *REC114* from the set of genes that induce double-strand DNA breaks, *SLX4* from the crossover I pathway, *MMS4* (also known as *EME1*) from the crossover II pathway, and *MSH3* from the set of mismatch-correction genes. These genes are not meiosis-specific, and not critical to their implicated processes. As is the case for *PMS1* and *PMS2*, we do not expect their absence to impact meiosis in Symbiodiniaceae.

Our observations of meiosis-specific and meiosis-related genes for *B. minutum* and *S. microadriaticum* are broadly consistent with earlier results by Chi *et al*.^9^, except for the absence of two genes in this study: *HOP2* in *B. minutum* and *PMS1* in *S. microadriaticum* based on the revised gene models. The *HOP2*-encoding sequence identified in *B. minutum* by Chi *et al*.^9^ failed to align to any putative protein homologs from SwissProt. The *PMS1*-encoding sequence identified in *S*. *microadriaticum* by Chi *et al*.^9^ aligned poorly to homologs from model organisms (mouse, human, *Dictyostelium discoideum*, *Arabidopsis thaliana*, yeasts *S. cerevisiae* and *S. pombe*) with a mismatch of 67% over 146 parsimony-informative sites. These results suggest that the previously identified genes are highly fragmented, or false positives. Liu *et al*.^10^ searched for meiosis-associated genes in *C. goreaui* and *F. kawagutii*, and found many more candidates than Chi *et al*.^9^. These likely include false positives, especially among genes that share high sequence identity such as the different *MSH*s and *SMC*s; for instance, a candidate protein for SLX1 may have been misannotated as SLX4. To make a stronger case for gene presence, we constructed single-gene phylogeny trees as described in Chi *et al.*^9^. Genes reported by Liu *et al.*^10^ to be present in *S. microadriaticum*, *B. minutum*, *C. goreaui*, and *F. kawagutii* but completely absent here based on revised gene models from Chen *et al.*^26^ are: *HOP1*, *ZIP1*, *REC8*, *SMC5*, *SMC6*, *RAD17*, *SLX4*, *MMS4*, and *MSH3*.

We only identified nine sex-associated genes in the *F. kawagutii* predicted gene set, but Morse^27^ found nine additional genes (*MND1*, *MLH1*, *MSH2*, *MSH3*, *MSH4*, *MSH5*, *RAD51A*, *SGS1*, and *SMC3*) using one-way BLAST searches from the *F. kawagutii* transcriptome data. Our putative candidates for the additional nine that Morse^27^ found did not fit our criteria (i.e., BLASTP hits with *e*-value < 10^−3^ and mutual coverage $\ge$25% against query sequences) and were thus considered absent.

For isolates for which genome data are not available (Table 1), absence of genes (based on searches using only transcriptome data) may represent false negatives. For instance, expression level of these genes may have been too low or likely not expressed under the conditions for which the transcript data were generated. This is likely the case for *Cladocopium* sp. Md and *Cladocopium* sp. C3k, in which completeness of transcriptome data based on Benchmarking Universal Single-Copy Orthologs (BUSCO) was 42.1% and 22.2% respectively (Fig. 1).

**Table 3:** Enriched GO terms (Biological Process) annotated among the combined predicted proteins of *S. microadriaticum*, *S. tridacnidorum*, *S. natans*, *B. minutum*, *F. kawagutii*, *C. goreaui*, and *Cladocopium* sp. C92, based on proteins coded by (a) genes exhibiting strong codon preference, or (b) genes under neutral selection (as test set), against all combined predicted gene models from the seven genomes as background. For each GO identifier, the associated term and statistical significance (*p*-value; Fisher’s exact test) are shown. Each list is sorted by the *p*-values, from the smallest to the largest. See Supplementary Table S1 online for the top five enriched GO terms for each isolate.

Test set	GO.ID	Term	*p*-value
(a) Strong codon preference	GO:0019253	Reductive pentose-phosphate cycle	7.9 × 10^−19^
	GO:0006006	Glucose metabolic process	1.9 × 10^−10^
	GO:0015991	ATP hydrolysis coupled proton transport	7.0 × 10^−10^
	GO:0015986	ATP synthesis coupled proton transport	2.5 × 10^−9^
	GO:0006414	Translational elongation	2.6 × 10^−8^
	GO:0022900	Electron transport chain	2.3 × 10^−7^
	GO:0006096	Glycolytic process	9.6 × 10^−7^
	GO:0055114	Oxidation-reduction process	1.3 × 10^−5^
	GO:0006418	tRNA aminoacylation for protein translation	3.7 × 10^−5^
	GO:0009399	Nitrogen fixation	5.6 × 10^−5^
(b) Neutral selection	GO:0006278	RNA-dependent DNA biosynthetic process	<1 × 10^−30^
	GO:0032197	Transposition, RNA-mediated	<1 × 10^−30^
	GO:0006508	Proteolysis	<1 × 10^−30^
	GO:0090502	RNA phosphodiester bond hydrolysis, endonucleolytic	<1 × 10^−30^
	GO:0090501	RNA phosphodiester bond hydrolysis	<1 × 10^−30^
	GO:0044238	Primary metabolic process	<1 × 10^−30^
	GO:0015074	DNA integration	<1 × 10^−30^
	GO:0071704	Organic substance metabolic process	<1 × 10^−30^
	GO:0015969	Guanosine tetraphosphate metabolic process	6.0 × 10^−30^
	GO:0044237	Cellular metabolic process	2.7 × 10^−25^

**Figure 2:**
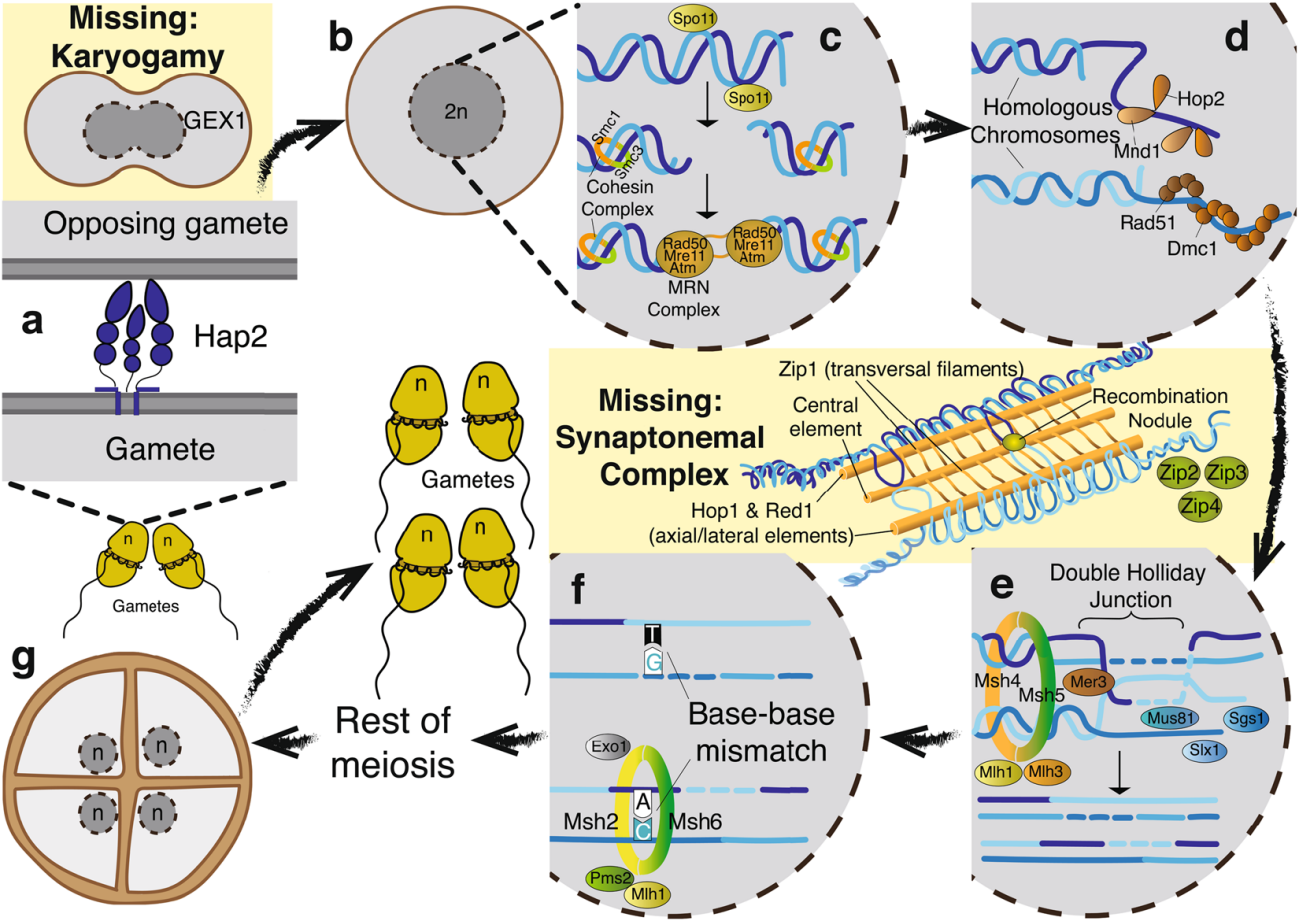
Hypothetical sexual stages of the life cycle in Symbiodiniaceae (ploidy n), showing all the relevant meiotic toolkit proteins. The missing complexes and processes, inferred from absence of genes, are shown against a yellow backdrop. The stages shown are (**a**) plasmogamy, (**b**) completion of gamete fusion, (**c**) formation of DNA double-strand break, (**d**) pairing of homologous chromosomes, (**e**) crossover II, (**f**) base-base mismatch correction, and (**g**) the end of meiosis, in which “tetrad” of daughter cells divides into four gametes. Stages (**c**) through (**f**) occur during prophase I of meiosis. The canonical synaptonemal complex forms around stage (**d**) until the end of prophase I. The “n” represents the haploid set of chromosomes. See text for detail.

We present the hypothetical sexual stages of the life cycle in Symbiodiniaceae in Fig. 2. During plasmogamy (Fig. 2a), Hap2 allows the “minus” mating type gamete to insert a loop into an opposing gamete^28^. The mechanism for karyogamy remains unclear because we did not recover *GEX1*. Meiosis begins in a diploid cell (Fig. 2b), following which Spo11 makes double-strand breaks in DNA (Fig. 2c). In canonical meiosis, the SC forms during synapsis and then degrades at the end of prophase I; this does not occur in Symbiodiniaceae (Fig. 2d,e,f). A pair of MRN complexes, composed of Rad50, Mre11, and Atm, tethers the broken ends of DNA strands together^29^. The Smc1-Smc3 heterodimer keeps sister chromosomes together, and the Hop2-Mnd1 complex then binds to DNA strands and searches for homologous chromosomes (Fig. 2d)^30^. Rad51 and Dmc1 assemble on double-strand breaks; Hop2-Mnd1 interacts with Rad51 and Dmc1, allowing single-strand invasion^31^. Crossover II then occurs: double Holliday junctions are resolved with the help of endonucleases Mus81 and Slx1, and helicases Sgs1 and Mer3 (Fig. 2e). The Msh4-Msh5 heterodimer, along with Mlh1 and Mlh3, keeps homologous chromosomes together. Finally, the Msh2-Msh6 complex recognises base-base mismatches in DNA (Fig. 2f), activated by Pms2 and Mlh1. Exo1 then excises the incorrect bases, and the DNA strands are further repaired^32^. At the end of prophase I, we presume the rest of meiosis proceeds similarly to other dinoflagellates (Fig. 2g); chromosomes stay condensed and are segregated via spindles that form outside the nucleus^33^.

### Are sex-associated genes under selection pressure?

We found that most sex-associated genes in Symbiodiniaceae have no codon usage preference, but trend towards “neutrality” (Table 3; see Supplementary Fig. S3, Supplementary Fig. S4 and Supplementary Fig. S5 online). This is similar to the meiotic genes in the yeast *Schizosaccharomyces pombe*, which showed an unbiased codon usage, in contrast to the highly biased ribosomal proteins^87^. From a total of 287 sex-associated genes found in this study, only seven (six *MER3*s and one *MND1*) show evidence of putative biased codon usage (i.e., distance of >25 units away from the diagonal line of neutrality plots in Supplementary Fig. S4 online). In addition, *MER3* in Symbiodiniaceae is highly diverged when compared to model organisms (e.g., see branch lengths in Supplementary Fig. S6 online), suggesting non-neutral evolution.

To determine which gene functions were undergoing selection, we annotated all the predicted gene models using Gene Ontology (GO) terms (Table 3). Genes with a strong codon preference were significantly enriched for the *reductive pentose-phosphate cycle* (GO:0019253), i.e., photosynthesis via C_3_ carbon fixation. Genes under neutral selection were enriched in several RNA-associated processes: *RNA-dependent DNA biosynthetic process* (GO:0006278), *RNA-mediated transposition* (GO:0032197), and *RNA phosphodiester bond hydrolysis* (GO:0090501 and GO:0090502). If taken together with another enriched process *DNA integration* (GO: 0015074), genes under neutral selection appear to be largely involved in the propagation of transposable elements. See Supplementary Table S1 online for the top five enriched GO terms for each isolate.

**Figure 3:**
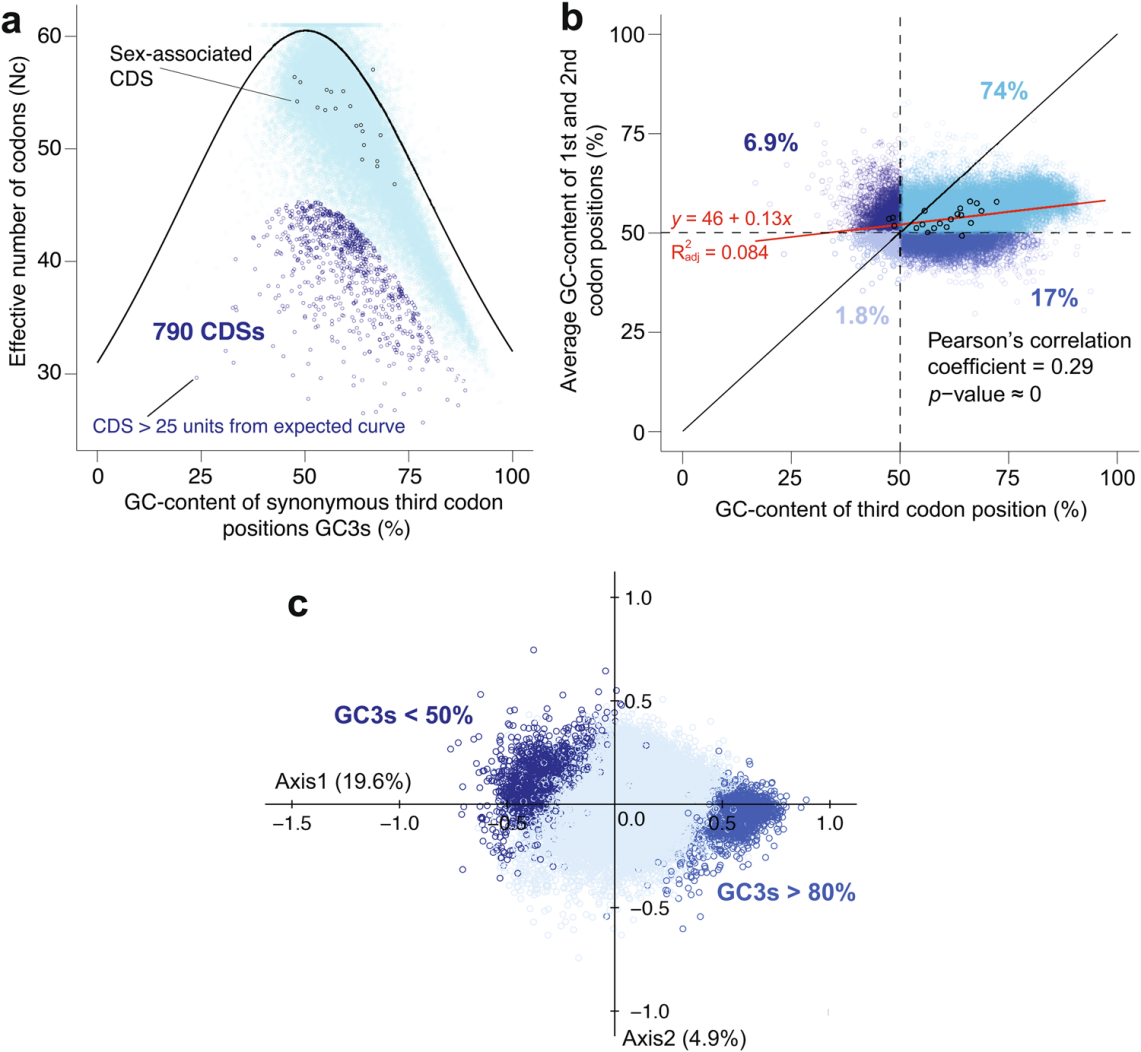
Codon usage trends of *Cladocopium goreaui*. Coding sequences (CDSs) were plotted according to their properties. (**a**) Effective number of codons (in *y*-axis) plotted against GC-content of synonymous third codon position (GC3s; in *x*-axis), in which the black curve represents the expected number of codons used under neutral selection. We consider CDSs at a distance >25 units below the expected curve to have a strong codon-usage preference. (**b**) Plot of neutrality, in which the diagonal line *y* = *x* represents neutrality. Genes closer to this line are under neutral selection. The trend line is shown in red; the percentage refers to the proportion of CDSs in each quadrant of GC-content. (**c**) Multi-variate correspondence analysis of relative synonymous codon usage, in which the *x* and *y* axes represent relative inertia. CDSs with GC3s-content below 50% and above 80% are highlighted in darker shades of blue.

Most isolates, including *P. glacialis* showed a similar trend of codon usage: overall GC-content of 50–58% with several hundred coding sequences (CDSs) exhibiting strong codon usage bias; see Supplementary Fig. S3, Supplementary Fig. S4 and Supplementary Fig. S5 online for codon usage trends for each isolate. Assuming that Symbiodiniaceae have a large effective population and that the effect of GC-biased gene conversion is small, most CDSs appear to be under non-neutral selection. The slight variation in codon usage observed for each isolate is attributed to the synonymous third codon position. For this analysis, we focused on *Cladocopium goreaui* as a representative of all isolates. Figure 3a shows the effective number of codons of CDSs in *C. goreaui* versus the GC-content of the synonymous third codon position (GC3s). The slope for the trend line for *C. goreaui* is 0.13 (i.e. <1.0), indicating that most of these CDSs may have been under selection for elevated GC-content in third codon positions. As shown in Fig. 3a, 790 CDSs show a distance of >25 units below the expected curve, indicating strong codon-usage preference; 74% of all CDSs in *C. goreaui* have >50% GC-content in all three codon positions (Fig. 3b). Figure 3c shows the multi-variate correspondence plot of relative synonymous codon usage, with each of the two axes representing the relative inertia that explains variation of the observed codon usage. Axis 1 explains 19.6% of variation in codon usage of all *C. goreaui* genes based on differences in GC3s.

## Discussion

Our results, based on the analysis of genome-scale data from a broadly sampled set of taxa, provide strong evidence for the lack of canonical SCs in Symbiodiniaceae. The lack of SCs has also been reported in other dinoflagellates. In species that have well-characterised sexual life cycles, such as in *Gymnodinium pseudopalustre*, chromosomes were “paired at a distance”^20^, suggesting that SCs are not needed for synapsis. One exception is the stretched-out chromosomes observed to form axial-loop structures in *Prorocentrum micans* during meiosis^34^, but they lacked the characteristic ladder-like organisation. These structures may represent dinoflagellate-specific synaptonemal complexes, although further confirmation is needed (Marie-Odile Soyer-Gobillard, *personal communication*, 26 August 2019). Such cytological observations do not exist for Symbiodiniaceae. Ciliates, which are basal alveolates, lack SCs in some lineages, while retaining “residual SC structures” in others (Table 2). Apicomplexans including *Plasmodium falciparum* are basal to dinoflagellates, but *P. falciparum* has canonical SCs and a near-complete meiotic gene inventory^35^. Therefore, our results suggest an independent loss of canonical SCs in dinoflagellates.

In canonical meiosis, SCs mediate class I crossovers. This pathway, favoured in *Arabidopsis* and mammals, involves ZMM proteins and MutL homologs that ensure crossovers happen at a distance from one another on a chromosome, resulting in a phenomenon known as interference. The tension produced from these distant points of crossovers allow the paired chromosomes to separate properly into daughter cells^36^. In contrast, the class II pathway recruits the Mus81-Mms4 complex, among others, to repair double-strand breaks at random positions^37^. Class II crossovers can also occur on SCs, usually near pericentric heterochromatin^38^. The highly repetitive sequences near the centromere are known to produce complex recombination intermediates that are thought to be better resolved by the class II pathway^39^.

In the SC-lacking *S. pombe* and ciliates, crossovers occur predominantly via the class II pathway^40,41^. We recovered *MUS81* and *SGS1* in Symbiodiniaceae, which encode the crossover junction endonuclease Mus81 and the DNA helicase Sgs1, respectively. These two proteins are essential for meiotic crossover in the SC-lacking ciliate *Tetrahymena thermophila*^41^. Therefore, we propose that Symbiodiniaceae also undergo class II crossovers in lieu of SC-mediated crossovers. To verify this class II crossover bias, we suggest comparing frequencies of different classes via immunostaining^39^.

The loss of SCs in dinoflagellates may be explained by two conditions. First, repeat sequences make up a substantial proportion (20–40%) of Symbiodiniaceae genomes^24^,^42^, and an even higher proportion (68%) in the genome of *Polarella glacialis*^43^, a closely related, earlier-diverging sister lineage. Second, dinoflagellates including Symbiodiniaceae have permanently condensed chromosomes. Although genome data from other more-anciently diverged dinoflagellates remain lacking, these conditions represent key idiosyncratic genome features in dinoflagellates^44^. We speculate that the tension provided by class I crossovers is not necessary for chromosome separation and therefore, SCs became expendable. We hypothesise that this loss started with the loss of function of *ZIP1* which encodes the transversal filaments (the axial elements are encoded by *HOP1* and *RED1*, which are still present in SC-lacking *S*. *pombe*^45^). Our hypotheses cannot explain the loss of SCs seen in some ciliates however, because they possess canonical chromosome architecture.

Talbert and Henikoff^46^ theorise that cell size increase in dinoflagellates during the Eocene due to warming temperatures led to genome size expansion. Together with the gain of dinoflagellate viral nucleoproteins, they propose that novel ways of packaging chromosomes arose in dinoflagellates. Extending this study to more basal dinoflagellates and alveolates will help reveal if this loss coincides with the appearance of permanently condensed chromosomes and an increase in repetitive elements^47^.

Diatoms also lack genes encoding canonical SCs; SC-like structures observed in some species suggest that other unidentified proteins may have replaced the SC^48^. Both cytological and *in silico* analysis demonstrated the lack of SCs in the fungus *Ustilago maydis*^49,50^. Similarly, SCs are completely absent in the fission yeast *S. pombe*; they instead produce single-lined linear elements. On the other hand, spermatogenesis in male fruit flies involves interlocked homologous chromosomes called bivalents that are physically sequestered into pockets of the prophase nucleus, leading to achiasmate meiosis^51^. Without cytological evidence, we cannot rule out the possibility that there may be such unidentified SC substitutes in Symbiodiniaceae. We also cannot rule out a parasexual process such as that in the fungus *Candida albicans*, in which a fusion of haploids undergoes recombination and chromosome loss until it is haploid again^52^. Many of the genes in the meiotic toolkit also play alternative roles in non-meiotic processes; for instance, *MND1* that repairs double-strand breaks in meiosis is also involved in telomere maintenance^53^. Therefore, the presence of the toolkit genes alone cannot prove an organism is sexual.

Identification of the *HAP2* gene using our approach suggests the existence of Symbiodiniaceae gametes. Questions about other characteristics, however, remain: are these gametes isogamous mastigotes as previous research suggests, and what are their mating types? Immunostaining of the Hap2 protein at different life cycle stages may prove useful in answering these questions.

The codon usage trends seen here corroborate results from earlier studies^54,55^, and agree with general codon usage in dinoflagellates. The Symbiodiniaceae and dinoflagellates studied thus far have an overall GC-content >50% in their coding regions, with the third codon GC-content varying more than the first or second, and the variation of codon bias within each species or isolate being correlated with the GC-content of the synonymous third codon position (GC3s)^56^. Hypotheses that may explain these findings are: (a) Symbiodiniaceae underwent selection pressure whereby high GC3s was favoured, or (b) as one would expect in mammals, many genes underwent concerted evolution which led to GC-biased gene conversion^57^. In any case, meiotic recombination likely drives these codon usage trends^14^, supporting the hypothesis that Symbiodiniaceae are sexual. Our results also suggest that these species undergo non-canonical meiosis using the class II crossover pathway that bypasses SC formation.

## Methods

### Dataset

We collected transcriptomes used in González-Pech *et al*.^55^. Original sources are listed in Table 1. Incomplete ORFs were removed from transcriptomes, then translated using EMBOSS transeq^58^. Proteins encoded by plastid and mitochondrial genes were removed using BLASTP^59^ searches against the RefSeq database (mitochondrion and plastid; release 75) following González-Pech *et al*.^55^. For the six species *Symbiodinium microadriaticum*, *Symbiodinium tridacnidorum*, *Breviolum minutum*, *Fugacium kawagutii*, *Cladocopium* sp. C92, and *Cladocopium goreui*, we used predicted gene models from Chen *et al*.^26^. To assess the completeness of each dataset, we searched for the alveolata_odb10 BUSCO^60^ genes using BLASTP (*e*-value < 10^–5^).

### Searching for homologous sequences

The relevant protein sequences available in the SwissProt database (release 2019_05) were used as queries to search against our dataset for putative homologs. Accession numbers of queries are listed in Supplementary Table S2 online. For genes that were very divergent, *Symbiodinium microadriaticum*^61^ and apicomplexan sequences from TrEMBL were used as queries. We chose a total of 42 genes, a combination of genes analysed in Chi *et al*.^9^ and Hofstatter *et al*.^2^. Genes that were peripheral players (e.g., *RAD52*, which mediates *RAD51* in homologous recombinational repair), or genes that did not have close homologs outside of metazoans (e.g., *BRCA1*, *BRCA2*) were excluded from our analysis. Hits with *e*-value < 10^−3^ and mutual coverage $\ge$25% were selected as candidates.

### Validation of candidate sequences

Identified homologs were further verified using a phylogenetic approach. Here, candidate sequences from each gene were first aligned to the query sequences, other SwissProt homologs, and homologs from closely-related taxa using MAFFT v7^62^ with the --auto setting. Alignments were trimmed using either trimAL v1.2^63^ with the -automated1 algorithm or BMGE v1.12^64^ with gap cut-off -g 0.4. Single-gene phylogenetic trees were constructed using IQ-TREE ModelFinder Plus using ultrafast bootstrap of 1000 replicates^65–67^. Putative homologs were verified if (a) they branched together with other dinoflagellates or alveolates, and (b) had branch lengths similar to those of queries. These sequences were then annotated by KofamScan^68^ using the KOfam eukaryotic database. The KOfam annotation of these genes are listed in Supplementary Table S3 online. The predicted gene models and protein sequences are available at 10.14264/uql.2020.483. Many Mnd1 candidates from the BLASTP step did not have corresponding KOfam hits to Mnd1, but this may be because Symbiodiniaceae sequences are very divergent.

### Codon usage trends

The effective number of codons, GC3s, and GC-content of each codon position for each CDS in the dataset were obtained using CodonW v1.3 (http://codonw.sourceforge.net/culong.html) and EMBOSS cusp^58^. Relative synonymous codon usage was used for correspondence analysis. Plots were generated using the R package ggpubr version 0.2.2 (https://cran.r-project.org/package=ggpubr). For transcriptome-alone isolates, we assumed 1 transcript = 1 CDS, as we did not have information on gene splicing from these data.

### Gene Ontology (GO) enrichment analysis

We performed GO enrichment analysis independently for each of the seven species: *S. microadriaticum*, *S. tridacnidorum*, *S. natans*, *B. minutum*, *F. kawagutii*, *C. goreaui*, and *Cladocopium* sp. C92 (Supplementary Table S1 online). For each species, we first used the predicted proteins as query and searched against the SwissProt database (release 2019_11; BLASTP, *E* ≤ 10^−5^) for putative homologs. Each protein sequence was then annotated based on the top SwissProt hit, and the associated GO terms to this hit were recovered using two in-house Python scripts (https://github.com/TimothyStephens/Annotate_GOterms_from_BLAST), following UniProt-GOA mapping (release 2019_05). Using the R package topGO version 2.36.0 (https://bioconductor.org/packages/topGO/), the enrichment analysis for GO terms associated with Biological Process was conducted using Fisher’s exact test. Independently for each species, a comparison was conducted for (a) proteins coded by genes exhibiting strong codon preference, and for (b) proteins coded by genes under neutral selection, each against all predicted proteins in the corresponding genome as background. Using combined, predicted proteins from all seven species, a comparison was also conducted independently for (a) proteins coded by genes exhibiting a strong codon preference, and for (b) proteins coded by genes under neutral selection, each against all predicted proteins from the seven genomes as background; these results are shown in Table 3. In total, our approach yielded 126,979 GO-annotated proteins, 2,400 proteins coded by genes exhibiting strong codon preference, and 47,422 proteins coded by genes under neutral selection (i.e., at a distance <5% from the diagonal line of *y* = *x* in Fig. 3b).

## Supplementary information

Supplementary Information.

## Data Availability

All gene models and protein sequences used in this study are available at 10.14264/uql.2020.483, except for the predicted protein sequences of *Polarella glacialis* and *Symbiodinium tridacnidorum* (hybrid genome assemblies) that were obtained from Stephens *et al*.^43^ and González-Pech *et al*.^24^ respectively.

## References

[1] Speijer D, Lukeš J, Eliáš M (2015). Sex is a ubiquitous, ancient, and inherent attribute of eukaryotic life. Proc. Natl. Acad. Sci. USA.

[2] Hofstatter PG, Brown MW, Lahr DJG (2018). Comparative genomics supports sex and meiosis in diverse amoebozoa. Genome Biol. Evol..

[3] Figueroa RI, Dapena C, Bravo I, Cuadrado A (2015). The hidden sexuality of *Alexandrium minutum*: An example of overlooked sex in dinoflagellates. PLos One.

[4] Price DC, Bhattacharya D (2017). Robust Dinoflagellata phylogeny inferred from public transcriptome databases. J. Phycol..

[5] Freudenthal HD (1962). *Symbiodinium* gen. nov. and *Symbiodinium microadriaticum* sp. nov., a zooxanthella: taxonomy, life cycle, and morphology. J. Protozool..

[6] Hughes TP (2018). Global warming transforms coral reef assemblages. Nature.

[7] LaJeunesse TC (2001). Investigating the biodiversity, ecology, and phylogeny of endosymbiotic dinoflagellates in the genus *Symbiodinium* using the ITS region: In search of a “species” level marker. J. Phycol..

[8] Brian JI, Davy SK, Wilkinson SP (2019). Multi-gene incongruence consistent with hybridisation in *Cladocopium* (Symbiodiniaceae), an ecologically important genus of coral reef symbionts. PeerJ.

[9] Chi J, Parrow MW, Dunthorn M (2014). Cryptic sex in *Symbiodinium* (Alveolata, Dinoflagellata) is supported by an inventory of meiotic genes. J. Eukaryot. Microbiol..

[10] Liu H (2018). *Symbiodinium* genomes reveal adaptive evolution of functions related to coral-dinoflagellate symbiosis. Commun. Biol..

[11] Fitt WK, Trench RK (1983). The relation of diel patterns of cell division to diel patterns of motility in the symbiotic dinoflagellate *Symbiodinium microadriaticum* Freudenthal in culture. New Phytol..

[12] Hadany L, Comeron JM (2008). Why are sex and recombination so common?. Ann. N. Y. Acad. Sci..

[13] Pouyet F, Mouchiroud D, Duret L, Sémon M (2017). Recombination, meiotic expression and human codon usage. elife.

[14] Webster MT, Hurst LD (2012). Direct and indirect consequences of meiotic recombination: implications for genome evolution. Trends Genet..

[15] Loidl J (2016). Conservation and variability of meiosis across the eukaryotes. Annu. Rev. Genet..

[16] San-Segundo PA, Roeder GS (1999). Pch2 links chromatin silencing to meiotic checkpoint control. Cell.

[17] Lynn A, Soucek R, Börner GV (2007). ZMM proteins during meiosis: crossover artists at work. Chromosome Res..

[18] Fédry J (2017). The ancient gamete fusogen Hap2 is a eukaryotic class II fusion protein. Cell.

[19] Ning J (2013). Comparative genomics in *Chlamydomonas* and *Plasmodium* identifies an ancient nuclear envelope protein family essential for sexual reproduction in protists, fungi, plants, and vertebrates. Genes Dev..

[20] von Stosch HA (1973). Observations on vegetative reproduction and sexual life cycles of two freshwater dinoflagellates, *Gymnodinium pseudopalustre* Schiller and *Woloszynskia apiculata* sp. nov. Br. Phycol..

[21] Wilcox LW, Wedemayer GJ, Graham LE (1982). *Amphidinium cryophilum* sp. nov. (dinophyceae) a new freshwater dinoflagellate. II. Ultrastructure. J. Phycol..

[22] Lindberg K, Moestrup Ø, Daugbjerg N (2005). Studies on woloszynskioid dinoflagellates I: *Woloszynskia coronata* re-examined using light and electron microscopy and partial LSU rDNA sequences, with description of *Tovellia* gen. nov. and *Jadwigia* gen. nov. (Tovelliaceae fam. nov.). Phycologia.

[23] Grishaeva, T. M. & Bogdanov, Y. F. Conservation and variability of synaptonemal complex proteins in phylogenesis of eukaryotes. *Int. J. Evol. Biol*., 856230 (2014). 10.1155/2014/856230 PMC4132317 25147749

[24] González-Pech, R. A. *et al*. Structural rearrangements drive extensive genome divergence between symbiotic and free-living *Symbiodinium*. *bioRxiv*, 783902 (2019).

[25] Hofstatter PG, Lahr DJG (2019). All eukaryotes are sexual, unless proven otherwise. BioEssays.

[26] Chen Y, González-Pech RA, Stephens TG, Bhattacharya D, Chan CX (2020). Evidence that inconsistent gene prediction can mislead analysis of dinoflagellate genomes. J. Phycol..

[27] Morse D (2019). A transcriptome-based perspective of meiosis in dinoflagellates. Protist.

[28] Baquero E, Fedry J, Legrand P, Krey T, Rey FA (2019). Species-specific functional regions of the green alga gamete fusion protein Hap2 revealed by structural studies. Structure.

[29] Alsbeih, G. MRE11A gene mutations responsible for the rare ataxia telangiectasia-like disorder in *Human Genetic Diseases* (ed. Plaseska-Karanfilska, D.) 79–90 (InTech Open, 2011).

[30] Zhao W (2014). Mechanistic insights into the role of Hop2-Mnd1 in meiotic homologous DNA pairing. Nucleic Acids Res..

[31] Vignard J (2007). The interplay of RecA-related proteins and the Mnd1-Hop2 complex during meiosis in *Arabidopsis thaliana*. PLoS Genet..

[32] Wei K, Kucherlapati R, Edelmann W (2002). Mouse models for human DNA mismatch-repair gene defects. Trends Mol. Med..

[33] Fukuda, Y. & Suzaki, T. Unusual features of dinokaryon, the enigmatic nucleus of dinoflagellates in *Marine Protists: Diversity and Dynamics* (eds Ohtsuka, S. *et al*.) 23–45 (Springer Japan, 2015).

[34] Soyer-Gobillard M-O, Bhaud Y, Hilaire D (2002). New data on mating in an autotrophic dinoflagellate, *Prorocentrum micans* Ehrenberg. Vie et Milieu.

[35] Lee AH, Symington LS, Fidock DA (2014). DNA repair mechanisms and their biological roles in the malaria parasite *Plasmodium falciparum*. Microbiol. Mol. Biol. Rev..

[36] Nambiar M, Chuang Y-C, Smith GR (2019). Distributing meiotic crossovers for optimal fertility and evolution. DNA Repair.

[37] Higgins JD, Buckling EF, Franklin FCH, Jones GH (2008). Expression and functional analysis of AtMUS81 in *Arabidopsis* meiosis reveals a role in the second pathway of crossing-over. Plant J..

[38] Demirci S (2017). Distribution, position and genomic characteristics of crossovers in tomato recombinant inbred lines derived from an interspecific cross between *Solanum lycopersicum* and *Solanum pimpinellifolium*. Plant J..

[39] Anderson LK (2014). Combined fluorescent and electron microscopic imaging unveils the specific properties of two classes of meiotic crossovers. Proc. Natl. Acad. Sci. USA.

[40] Chi J, Mahé F, Loidl J, Logsdon J, Dunthorn M (2013). Meiosis gene inventory of four ciliates reveals the prevalence of a synaptonemal complex-independent crossover pathway. Mol. Biol. Evol..

[41] Lukaszewicz A, Howard-Till RA, Loidl J (2013). Mus81 nuclease and Sgs1 helicase are essential for meiotic recombination in a protist lacking a synaptonemal complex. Nucleic Acids Res..

[42] González-Pech, R. A. *et al*. Genomes of Symbiodiniaceae reveal extensive sequence divergence but conserved functions at family and genus levels. *bioRxiv*, 800482 (2019).

[43] Stephens, T. G. *et al*. Genomes of the dinoflagellate *Polarella glacialis* encode tandemly repeated single-exon genes with adaptive functions. *BMC Biology* **18,** 56 (2020). 10.1186/s12915-020-00782-8 PMC7245778 32448240

[44] Lin S (2011). Genomic understanding of dinoflagellates. Res. Microbiol..

[45] Lorenz A (2004). *S. pombe* meiotic linear elements contain proteins related to synaptonemal complex components. J. Cell Sci..

[46] Talbert PB, Henikoff S (2012). Chromatin: packaging without nucleosomes. Curr. Biol..

[47] Gornik SG, Hu I, Lassadi I, Waller RF (2019). The biochemistry and evolution of the dinoflagellate nucleus. Microorganisms.

[48] Patil S (2015). Identification of the meiotic toolkit in diatoms and exploration of meiosis-specific SPO11 and RAD51 homologs in the sexual species *Pseudo-nitzschia* multistriata and *Seminavis robusta*. BMC Genomics.

[49] Fletcher HL (1981). A search for synaptonemal complexes in *Ustilago maydis*. J. Cell Sci..

[50] Donaldson ME, Saville BJ (2008). Bioinformatic identification of *Ustilago maydis* meiosis genes. Fungal Genet. Biol..

[51] Vazquez J, Belmont AS, Sedat JW (2002). The dynamics of homologous chromosome pairing during male *Drosophila* meiosis. Curr. Biol..

[52] Bennett RJ, Johnson AD (2003). Completion of a parasexual cycle in *Candida albicans* by induced chromosome loss in tetraploid strains. EMBO J..

[53] Maciver, S. K. Ancestral eukaryotes reproduced asexually, facilitated by polyploidy: a hypothesis. *BioEssays ***41,** 1900152 (2019). 10.1002/bies.201900152 31667871

[54] Bayer T (2012). *Symbiodinium* transcriptomes: genome insights into the dinoflagellate symbionts of reef-building corals. Plos One.

[55] González-Pech RA, Ragan MA, Chan CX (2017). Signatures of adaptation and symbiosis in genomes and transcriptomes of *Symbiodinium*. Sci. Rep..

[56] Williams E, Place A, Bachvaroff T (2017). Transcriptome analysis of core dinoflagellates reveals a universal bias towards “GC” rich codons. Mar. Drugs.

[57] Duret L, Galtier N (2009). Biased gene conversion and the evolution of mammalian genomic landscapes. Annu. Rev. Genomics Hum. Genet..

[58] Rice P, Longden I, Bleasby A (2000). EMBOSS: The European Molecular Biology Open Software Suite. Trends Genet..

[59] Altschul SF, Gish W, Miller W, Myers EW, Lipman DJ (1990). Basic local alignment search tool. J. Mol. Biol..

[60] Simão FA, Waterhouse RM, Ioannidis P, Kriventseva EV, Zdobnov EM (2015). BUSCO: assessing genome assembly and annotation completeness with single-copy orthologs. Bioinformatics.

[61] Aranda M (2016). Genomes of coral dinoflagellate symbionts highlight evolutionary adaptations conducive to a symbiotic lifestyle. Sci. Rep..

[62] Katoh K, Standley DM (2013). MAFFT multiple sequence alignment software version 7: improvements in performance and usability. Mol. Biol. Evol..

[63] Capella-Gutiérrez S, Silla-Martínez JM, Gabaldón T (2009). trimAl: a tool for automated alignment trimming in large-scale phylogenetic analyses. Bioinformatics.

[64] Criscuolo A, Gribaldo S (2010). BMGE (Block Mapping and Gathering with Entropy): a new software for selection of phylogenetic informative regions from multiple sequence alignments. BMC Evol. Biol..

[65] Hoang DT, Chernomor O, von Haeseler A, Minh BQ, Vinh LS (2018). UFBoot2: improving the ultrafast bootstrap approximation. Mol. Biol. Evol..

[66] Kalyaanamoorthy S, Minh BQ, Wong TKF, von Haeseler A, Jermiin LS (2017). ModelFinder: fast model selection for accurate phylogenetic estimates. Nat. Meth..

[67] Nguyen L-T, Schmidt HA, von Haeseler A, Minh BQ (2015). IQ-TREE: a fast and effective stochastic algorithm for estimating maximum-likelihood phylogenies. Mol. Biol. Evol..

[68] Aramaki T (2020). KofamKOALA: KEGG ortholog assignment based on profile HMM and adaptive score threshold. Bioinformatics.

[69] LaJeunesse TC (2018). Systematic revision of Symbiodiniaceae highlights the antiquity and diversity of coral endosymbionts. Curr. Biol..

[70] Davies SW, Marchetti A, Ries JB, Castillo KD (2016). Thermal and pCO2 stress elicit divergent transcriptomic responses in a resilient coral. Front. Mar. Sci..

[71] Ladner JT, Barshis DJ, Palumbi SR (2012). Protein evolution in two co-occurring types of *Symbiodinium*: an exploration into the genetic basis of thermal tolerance in *Symbiodinium* clade D. BMC Evol. Biol..

[72] González-Pech RA, Vargas S, Francis WR, Wörheide G (2017). Transcriptomic resilience of the Montipora digitata holobiont to low pH. Front. Mar. Sci..

[73] Levin RA (2016). Sex, scavengers, and chaperones: transcriptome secrets of divergent symbiodinium thermal tolerances. Mol. Biol. Evol..

[74] Parkinson JE (2016). Gene expression variation resolves species and individual strains among coral-associated dinoflagellates within the genus Symbiodinium. Genome Biol. Evol..

[75] Xiang T, Nelson W, Rodriguez J, Tolleter D, Grossman AR (2015). *Symbiodinium* transcriptome and global responses of cells to immediate changes in light intensity when grown under autotrophic or mixotrophic conditions. Plant J..

[76] Shoguchi E (2013). Draft assembly of the *Symbiodinium minutum* nuclear genome reveals dinoflagellate gene structure. Curr. Biol..

[77] Shoguchi E (2018). Two divergent *Symbiodinium* genomes reveal conservation of a gene cluster for sunscreen biosynthesis and recently lost genes. BMC Genomics.

[78] Loidl J, Scherthan H (2004). Organization and pairing of meiotic chromosomes in the ciliate *Tetrahymena thermophila*. J. Cell Sci..

[79] Round FE, Crawford RM, Mann DG (1991). The diatoms: Biology & morphology of the genera. Taxon.

[80] Mann DG, Stickle AJ (1989). Meiosis, nuclear cyclosis, and auxospore formation in *Navicula* sensu stricto (Bacillariophyta). Br. Phycol..

[81] Manton I, Kowallik K, Stosch HA (1969). v. Observations on the fine structure and development of the spindle at mitosis and meiosis in a marine centric diatom (Lithodesmium undulatum). J. Microsc..

[82] Holloman WK, Schirawski J, Holliday R (2008). The homologous recombination system of Ustilago maydis. Fungal Genet. Biol..

[83] Kohli J, Bähler J (1994). Homologous recombination in fission yeast: absence of crossover interference and synaptonemal complex. Experientia.

[84] Egel-Mitani M, Olson LW, Egel R (1982). Meiosis in *Aspergillus nidulans*: another example for lacking synaptonemal complexes in the absence of crossover interference. Hereditas.

[85] Rasmussen SW (1973). Ultrastructural studies of spermatogenesis in *Drosophila melanogaster* meigen. Z Zellforsch Mikrosk Anat.

[86] Gilboa L, Lehmann R (2004). How different is Venus from Mars? The genetics of germ-line stem cells in *Drosophila* females and males. Development.

[87] Hiraoka, Y, Kawamata K., Haraguchi T. & Chikashige, Y. Codon usage bias is correlated with gene expression levels in the fission yeast *Schizosaccharomyces pombe*. *Genes* *Cells* **14**, 499-509 (2009). 10.1111/j.1365-2443.2009.01284.x 19335619

