# Supplementary material for: Sex in Symbiodiniaceae dinoflagellates: genomic evidence for independent loss of the canonical synaptonemal complex
Source: Sci Rep. 2020 Jun 17;10:9792. doi: 10.1038/s41598-020-66429-4 (PMC7299967; doi:10.1038/s41598-020-66429-4)
Supplement: Supplementary file 1 — Supplementary Information. [file 41598_2020_66429_MOESM1_ESM.pdf]

Supplementary Information

Sex in Symbiodiniaceae dinoflagellates: genomic evidence for independent loss of the canonical synaptonemal complex

Sarah Shah[1,2,3], Yibi Chen[1,2,3], Debashish Bhattacharya[4], and Cheong Xin Chan[1,2,3,*]

[1]Institute for Molecular Bioscience, The University of Queensland, Brisbane, QLD 4072, Australia

[2]School of Chemistry and Molecular Biosciences, The University of Queensland, Brisbane, QLD 4072, Australia

[3]Australian Centre for Ecogenomics, The University of Queensland, Brisbane, QLD 4072, Australia

[4]Department of Biochemistry and Microbiology, Rutgers University, New Brunswick, NJ 08901, U.S.A.

*Correspondence and requests for materials should be addressed to Cheong Xin Chan (c.chan1@uq.edu.au)

Supplementary Figures S1 through S6

Supplementary Tables S1 through S3

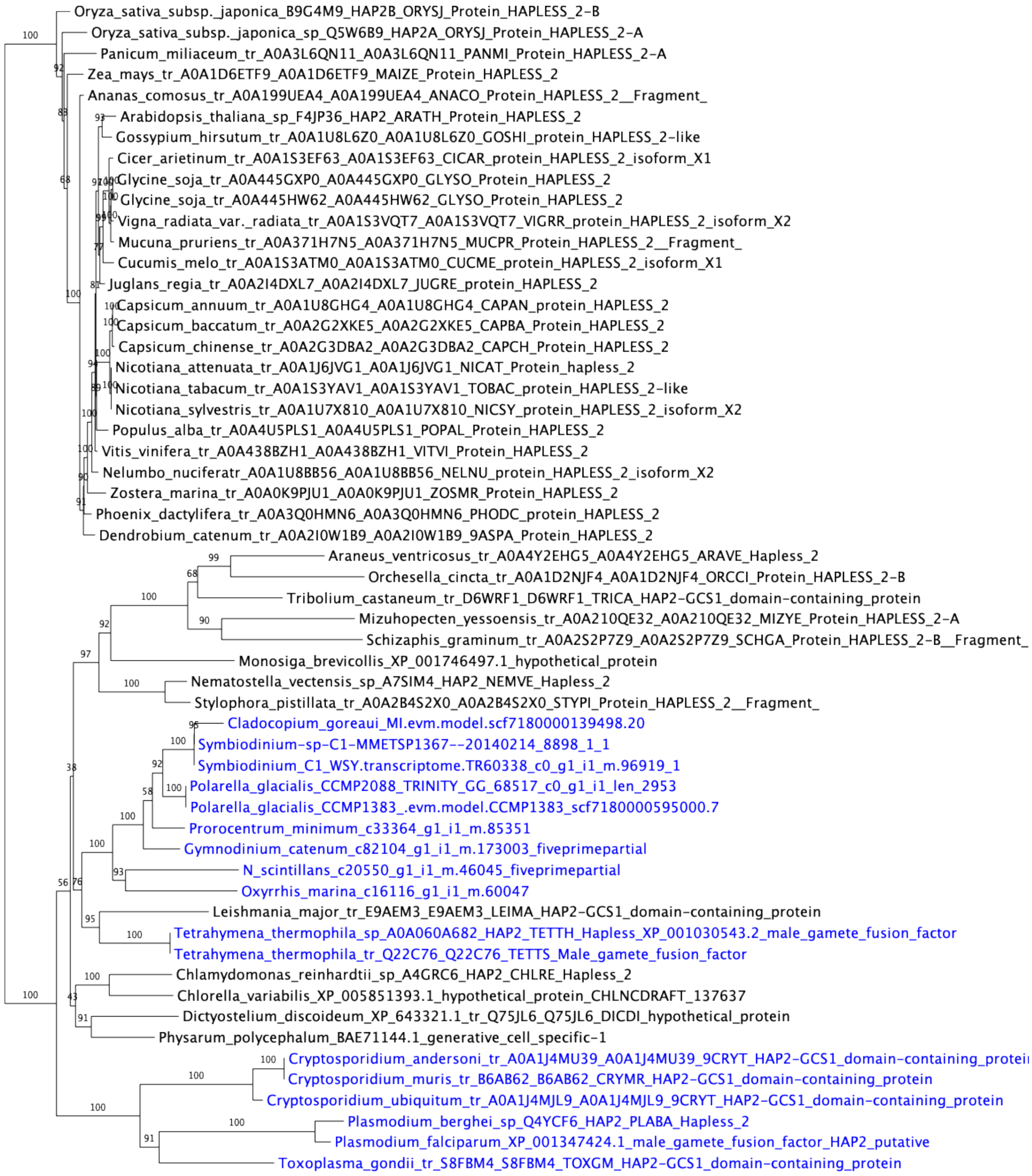

Supplementary Figure S1. Hap2 phylogeny for validation of putative homologs. Tree is rooted by plant Hap2 sequences. Sequences in blue are alveolate homologs. Maximum likelihood estimation using substitution model LG+R4 (selected by IQ-TREE ModelFinder Plus) for an alignment containing 344 parsimony-informative amino acid sites. Numbers above each branch refer to ultrafast bootstrap values from 1,000 replicates.

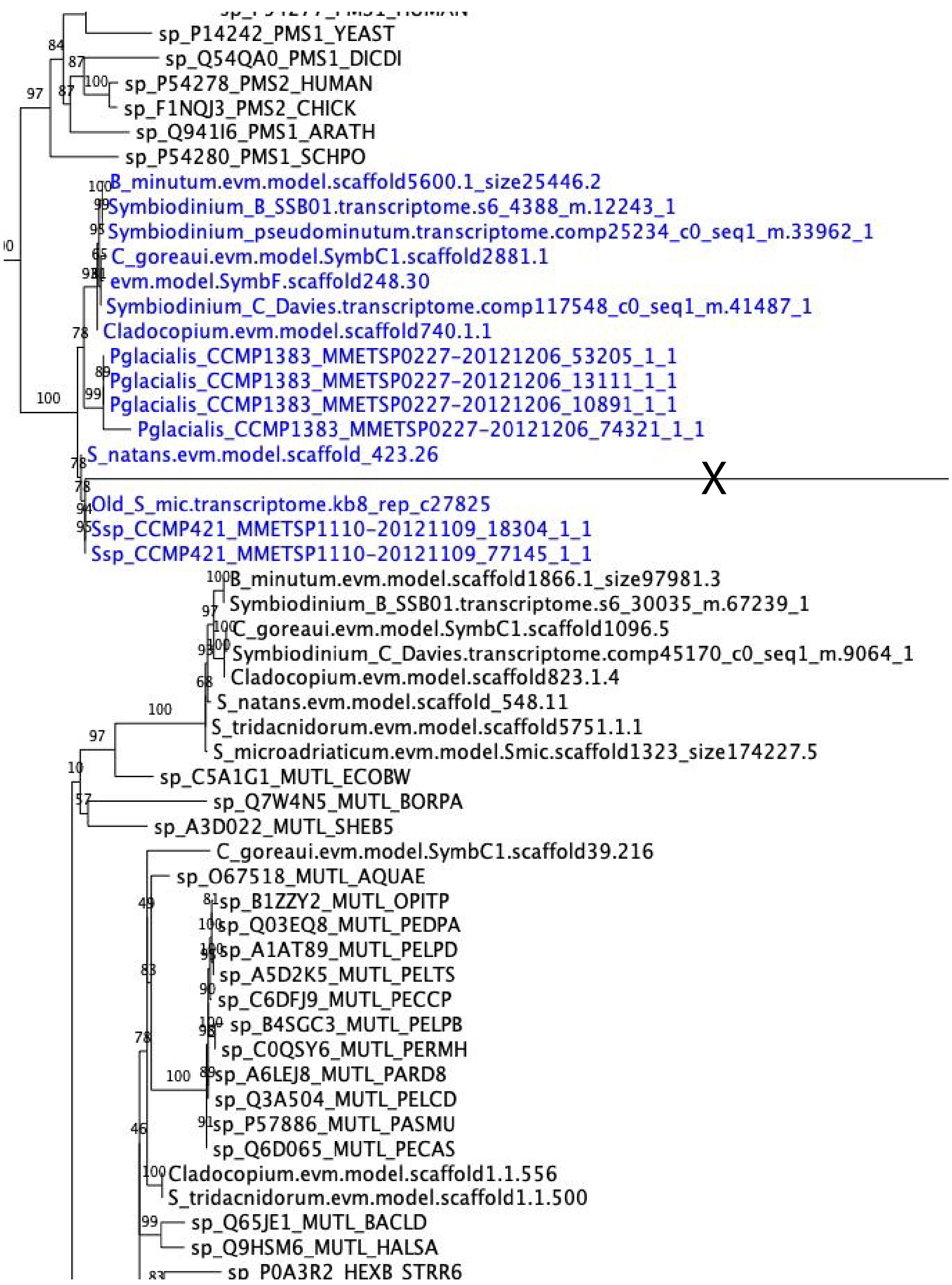

Supplementary Figure S2. Pms1 and Pms2 phylogeny for validation of putative homologs. Sequences in blue are Symbiodiniaceae and *P. glacialis* candidates. Maximum likelihood estimation using substitution model LG+R8 (selected by IQ-TREE ModelFinder Plus) for an alignment containing 84 parsimony-informative amino acid sites. The long branch indicated by X belonged to a Symbiodiniaceae sequence that was too divergent and was dropped from analysis.

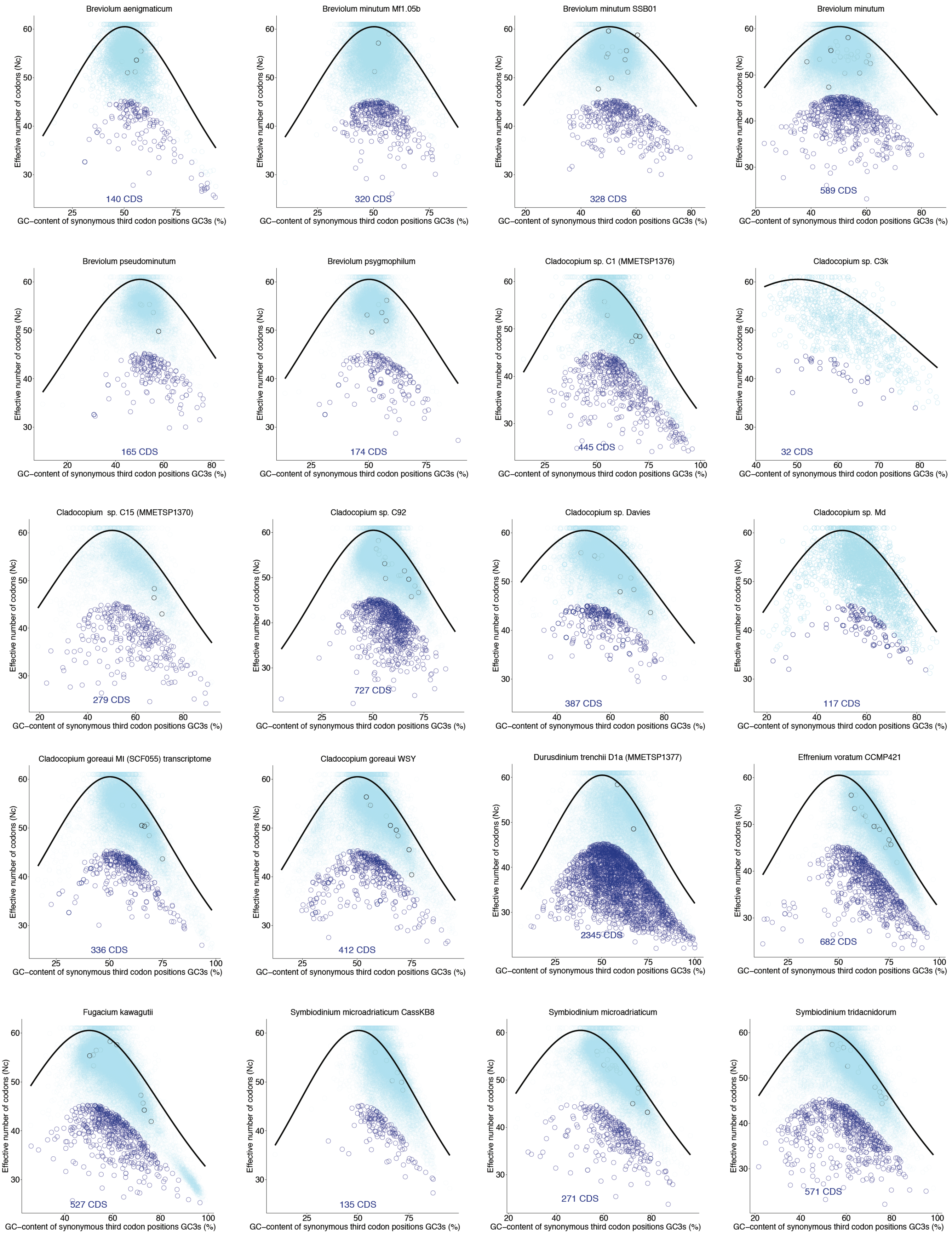

Supplementary Figure S3. Plots of effective number of codons (Nc) versus GC-content of synonymous third codon positions (GC3s) of coding sequences (CDS) of Symbiodiniaceae isolates.

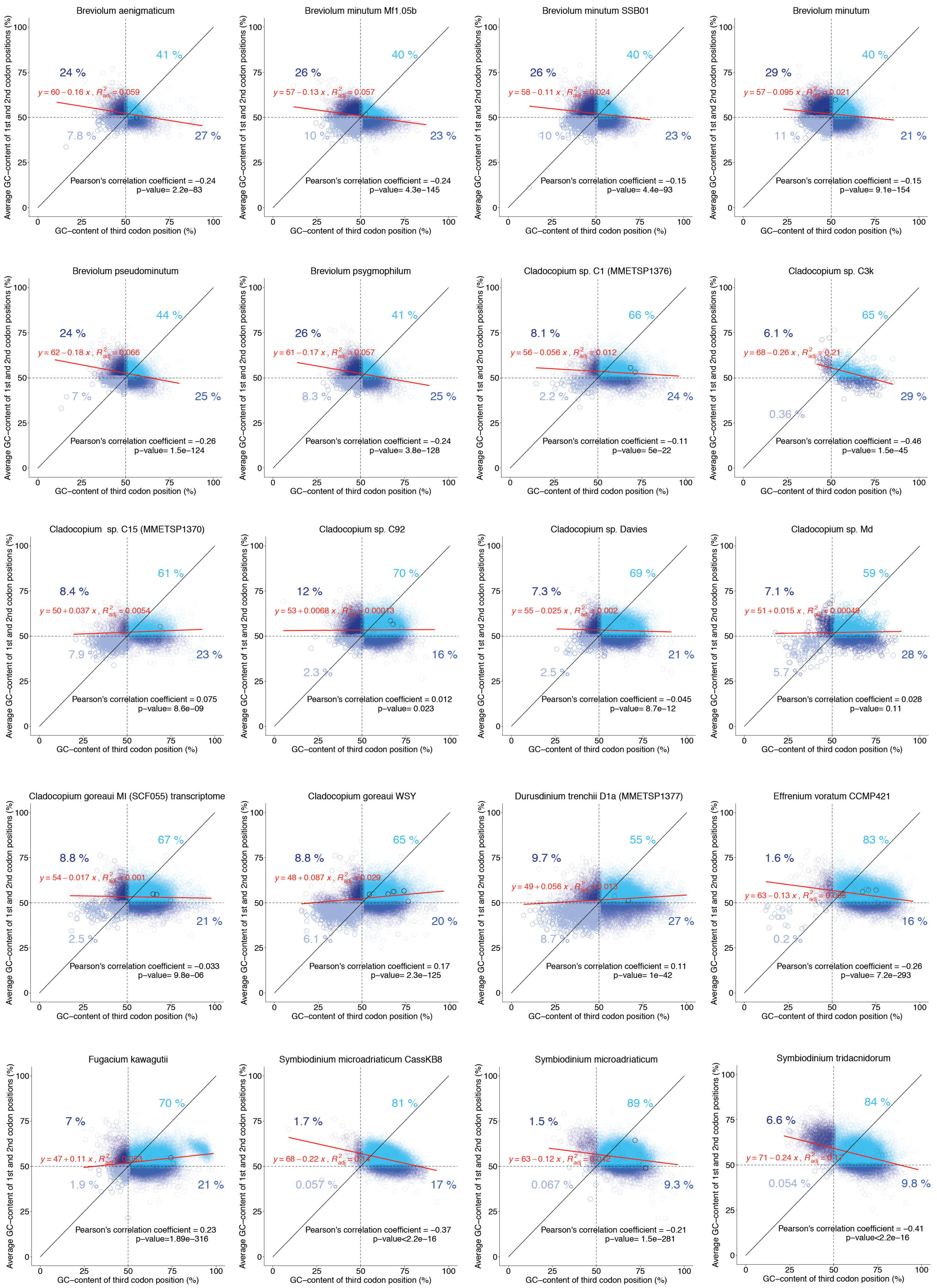

Supplementary Figure S4. Neutrality plots of Symbiodiniaceae CDS.

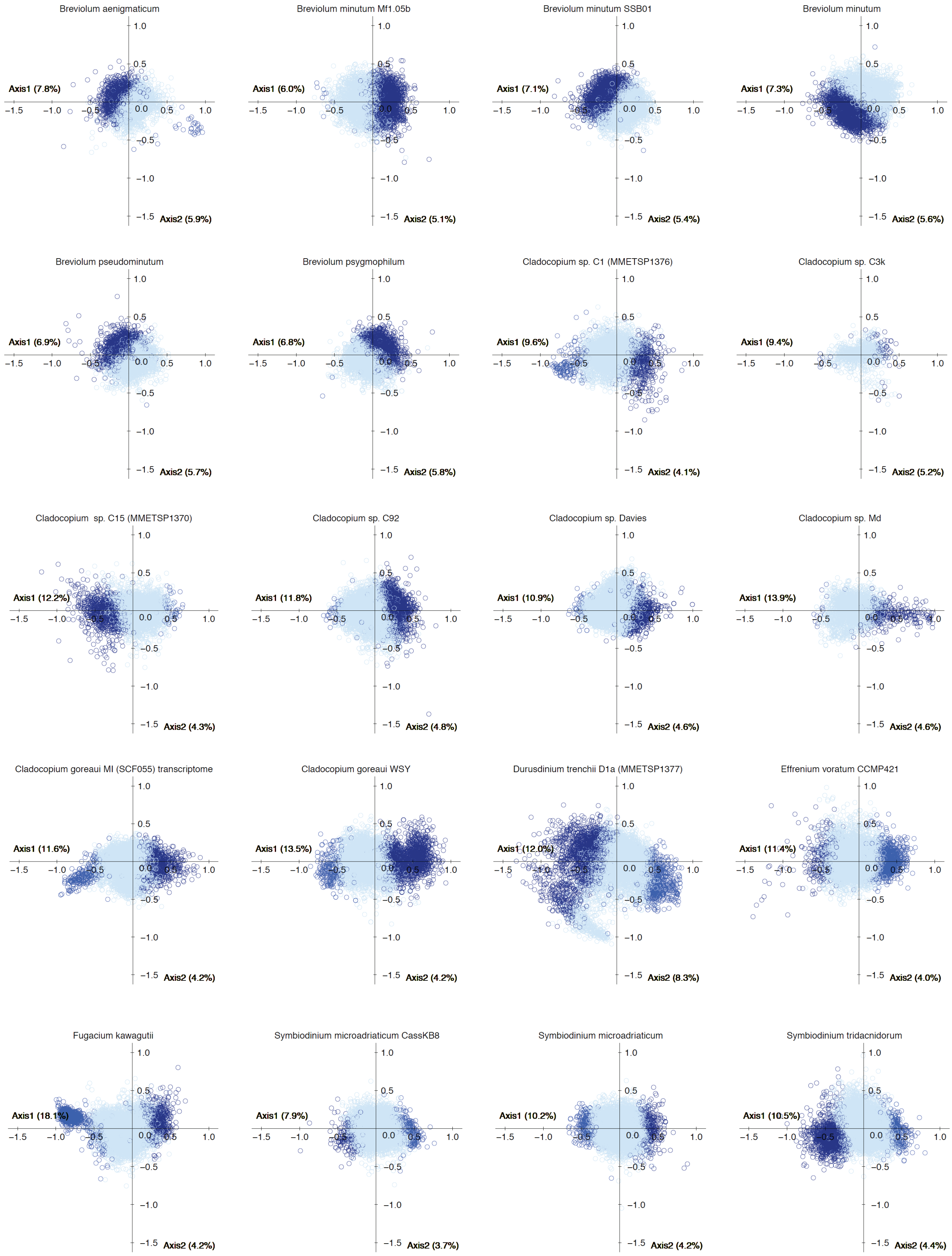

Supplementary Figure S5. Plots of multi-variate correspondence analysis of relative synonymous codon usage in Symbiodiniaceae.

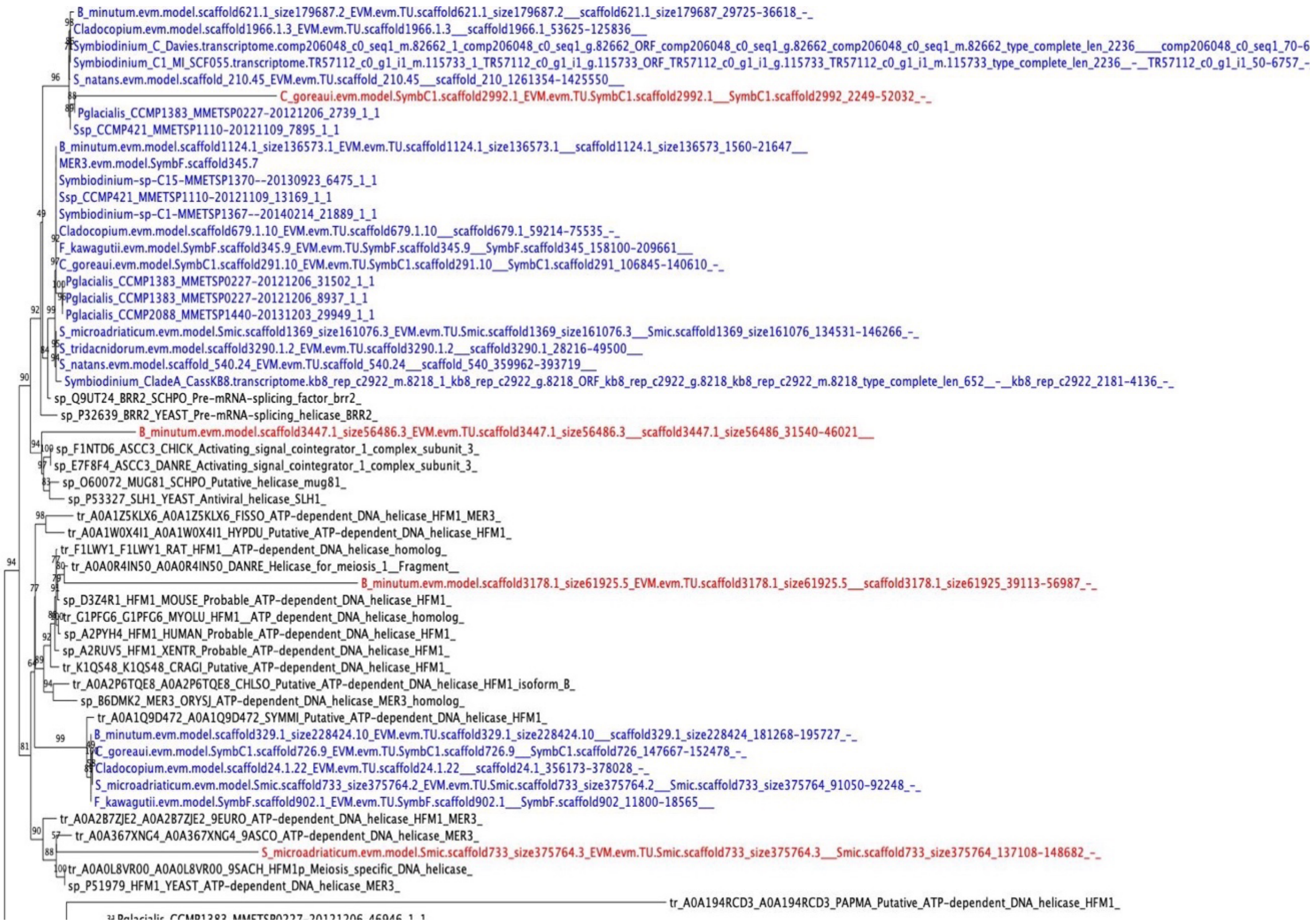

Supplementary Figure S6. Mer3 phylogeny for validation of putative homologs. Maximum likelihood estimation using substitution model LG+F+G4 (selected by IQ-TREE ModelFinder Plus) for an alignment containing 83 parsimony-informative amino acid sites. Symbiodiniaceae and *P. glacialis* candidate sequences are in blue. Those in red were too long-branching and were dropped out of the analysis.

Supplementary Table S1. Top ten GO enrichment biological processes for all seven species *S. microadriaticum*, *S. tridacnidorum*, *S. natans*, *B. minutum*, *F. kawagutii*, *C. goreaui*, and *Cladocopium* sp. C92, followed by the top five GO enrichment processes for each individual species.

Isolate	Test set	GO.ID	Term	Annotated	Significant	Expected	Fisher p-value
All seven isolates	Strong codon preference	GO:0019253	Reductive pentose-phosphate cycle	158	24	2.79	$7.9x10^{-19}$
		GO:0006006	Glucose metabolic process	1,058	43	18.67	$1.9x10^{-10}$
		GO:0015991	ATP hydrolysis coupled proton transport	248	19	4.38	$7.0x10^{-10}$
		GO:0015986	ATP synthesis coupled proton transport	611	29	10.78	$2.5x10^{-9}$
		GO:0006414	Translational elongation	762	31	13.45	$2.6x10^{-8}$
		GO:0022900	Electron transport chain	1,225	41	21.62	$2.3x10^{-7}$
		GO:0006096	Glycolytic process	1,090	35	19.24	$9.6x10^{-7}$
		GO:0055114	Oxidation-reduction process	11,865	229	209.39	$1.3x10^{-5}$
		GO:0006418	tRNA aminoacylation for protein translation	3,382	86	59.69	$3.7x10^{-5}$
		GO:0009399	Nitrogen fixation	133	9	2.35	$5.6x10^{-5}$
All seven isolates	Neutral selection	GO:0006278	RNA-dependent DNA biosynthetic process	2,910	1,364	1097.72	$< 1x10^{-30}$
		GO:0032197	Transposition, RNA-mediated	2,093	981	789.53	$< 1x10^{-30}$
		GO:0006508	Proteolysis	10,150	3,743	3828.82	$< 1x10^{-30}$
		GO:0090502	RNA phosphodiester bond hydrolysis, endonucleolytic	2,245	992	846.87	$< 1x10^{-30}$
		GO:0090501	RNA phosphodiester bond hydrolysis	4,278	1,888	1613.76	$< 1x10^{-30}$
		GO:0044238	Primary metabolic process	71,661	24,130	27032.24	$< 1x10^{-30}$
		GO:0015074	DNA integration	1,440	672	543.2	$< 1x10^{-30}$
		GO:0071704	Organic substance metabolic process	77,632	26,135	29284.64	$< 1x10^{-30}$
		GO:0015969	Guanosine tetraphosphate metabolic process	1,510	658	569.61	$6.0x10^{-30}$
		GO:0044237	Cellular metabolic process	75,201	25,155	28367.61	$2.7x10^{-25}$
Symbiodinium microadriaticum	Strong codon preference	GO:0019253	Reductive pentose-phosphate cycle	27	3	0.18	0.00074
		GO:0009399	Nitrogen fixation	28	3	0.19	0.00083
		GO:1903478	Actin filament bundle convergence involved in mitotic contractile ring assembly	12	2	0.08	0.00276
		GO:0006094	Gluconeogenesis	149	5	0.99	0.00319
		GO:0043039	tRNA aminoacylation	744	14	4.94	0.00525

	Neutral selection	GO:0006278	RNA-dependent DNA biosynthetic process	854	358	196.94	< $1x10^{-30}$
		GO:0032197	Transposition, RNA-mediated	688	296	158.66	< 1e-30
		GO:0090502	RNA phosphodiester bond hydrolysis, endonucleolytic	638	272	147.13	$6.10x10^{-29}$
		GO:0090501	RNA phosphodiester bond hydrolysis	1176	480	271.19	$4.90x10^{-18}$
		GO:0006508	Proteolysis	2148	655	495.34	$2.20x10^{-17}$
Symbiodinium tridacnidorum	Strong codon preference	GO:0019752	Carboxylic acid metabolic process	2934	74	50.19	0.00025
		GO:0039663	Membrane fusion involved in viral entry	34	4	0.58	0.0026
		GO:0019087	Transformation of host cell by virus	20	3	0.34	0.00455
		GO:0039545	Suppression by virus of host MAVS activity	20	3	0.34	0.00455
		GO:0039547	Suppression by virus of host TRAF activity	20	3	0.34	0.00455
	Neutral selection	GO:0006278	RNA-dependent DNA biosynthetic process	398	154	90.92	$5.10x10^{-13}$
		GO:0032197	Transposition, RNA-mediated	298	115	68.08	$5.50x10^{-10}$
		GO:0090501	RNA phosphodiester bond hydrolysis	658	231	150.31	$5.50x10^{-8}$
		GO:0090502	RNA phosphodiester bond hydrolysis, endonucelolytic	363	124	82.92	$4.60x10^{-7}$
		GO:0015969	Guanosine tetraphosphate metabolic process	300	106	68.53	$5.10x10^{-7}$
Breviolum minutum	Strong codon preference	GO:0006096	Glycolytic process	216	13	4.35	$3.20x10^{-5}$
		GO:0006414	Translational elongation	154	10	3.1	0.00014
		GO:0006006	Glucose metabolic process	212	14	4.27	0.00052
		GO:0006422	Aspartyl-tRNA aminoacylation	47	5	0.95	0.00075
		GO:0043419	Urea catabolic process	13	3	0.26	0.00092
	Neutral selection	GO:0008150	Biological process	22271	12419	12466.11	< $1x10^{-30}$
		GO:0000455	Enzyme-directed rRNA pseudouridine synthesis	67	52	37.5	0.00014
		GO:0071432	Peptide mating pheromone maturation involved in positive regulation of conjugation	13	13	7.28	0.00047
		GO:0030091	Protein repair	53	41	29.67	0.00079
		GO:0140053	Mitochondrial gene expression	301	193	168.48	0.00137
Fugacium kawagutii	Strong codon preference	GO:0019253	Reductive pentose-phosphate cycle	25	12	0.5	$1.00x10^{-14}$
		GO:0015986	ATP synthesis coupled proton transport	23	7	0.46	$2.00x10^{-7}$
		GO:0006006	Glucose metabolic process	76	10	1.51	$2.20x10^{-6}$
		GO:0022900	Electron transport chain	106	11	2.11	0.00015

		GO:0015991	ATP hydrolysis coupled proton transport	21	4	0.42	0.00069
	Neutral selection	GO:0019253	Reductive pentose-phosphate cycle	25	6	5.67	1.00x10^{-14}
		GO:0015986	ATP synthesis coupled proton transport	23	4	5.21	2.00x10^{-7}
		GO:0006006	Glucose metabolic process	76	13	17.23	2.20x10^{-6}
		GO:0022900	Electron transport chain	106	13	24.03	0.00015
		GO:0015991	ATP hydrolysis coupled proton transport	21	6	4.76	0.00069
Cladocopium goreaui	Strong codon preference	GO:0019253	Reductive pentose-phosphate cycle	14	4	0.24	6.80x10^{-5}
		GO:0006412	Translation	557	21	9.35	0.00043
		GO:1990443	Peptidyl-threonine autophosphorylation	4	2	0.07	0.00165
		GO:0015991	ATP hydrolysis coupled proton transport	17	3	0.29	0.00266
		GO:0048653	Anther development	5	2	0.08	0.00271
	Neutral selection	GO:0006552	Leucine catabolic process	9	8	2.19	8.70x10^{-5}
		GO:0010638	Positive regulation of organelle organization	137	57	33.38	0.00017
		GO:0031930	Mitochondria-nucleus signalling pathway	164	60	39.95	0.00029
		GO:0010019	Chloroplast-nucleus signalling pathway	164	60	39.95	0.00029
		GO:0006278	RNA-dependent DNA biosynthetic process	72	31	17.54	0.00037
Cladocopium sp. C92	Strong codon preference	GO:0008150	Biological process	22643	355	588.97	3.80x10^{-17}
		GO:0009987	Cellular process	18723	298	487.01	6.20x10^{-9}
		GO:0044238	Primary metabolic process	15288	243	397.66	5.50x10^{-6}
		GO:1901566	Organonitrogen compound biosynthetic process	5247	115	136.48	7.40x10^{-6}
		GO:1901564	Organonitrogen compound metabolic process	10613	194	276.06	8.70x10^{-5}
	Neutral selection	GO:0008150	Biological process	22643	9142	12970.06	< 1x10^{-30}
		GO:0006508	Proteolysis	2440	1144	1397.65	< 1x10^{-30}
		GO:0006278	RNA-dependent DNA biosynthetic process	927	505	530.99	< 1x10^{-30}
		GO:0032197	Transposition, RNA-mediated	706	381	404.4	< 1x10^{-30}
		GO:0090501	RNA phosphodiester bond hydrolysis	1313	665	752.09	< 1x10^{-30}

Supplementary Table S2. UniProtKB, NCBI, and trEMBL accession numbers of protein queries used for homology search.

Accession number	Description
sp|P14291|RED1_YEAST	Protein RED1 OS=Saccharomyces cerevisiae (strain ATCC 204508 / S288c) OX=559292 GN=RED1 PE=1 SV=1
sp|P31111|ZIP1_YEAST	Synaptonemal complex protein ZIP1 OS=Saccharomyces cerevisiae (strain ATCC 204508/S288c) OX=559292 GN=ZIP1 PE=1 SV=2
sp|B6DMK2|MER3_ORYSJ	ATP-dependent DNA helicase MER3 homolog OS=Oryza sativa subsp. japonica OX=39947 GN=MER3 PE=2 SV=1
sp|F4JP48|MSH4_ARATH	DNA mismatch repair protein MSH4 OS=Arabidopsis thaliana OX=3702 GN=MSH4 PE=2 SV=1
sp|F4JEP5|MSH5_ARATH	DNA mismatch repair protein MSH5 OS=Arabidopsis thaliana OX=3702 GN=MSH5 PE=2 SV=1
sp|Q6Q1P4|SMC1_ARATH	Structural maintenance of chromosomes protein 1 OS=Arabidopsis thaliana OX=3702 GN=SMC1 PE=2 SV=2
sp|Q56YN8|SMC3_ARATH	Structural maintenance of chromosomes protein 3 OS=Arabidopsis thaliana OX=3702 GN=SMC3 PE=2 SV=1
sp|Q9LFS8|SMC5_ARATH	Structural maintenance of chromosomes protein 5 OS=Arabidopsis thaliana OX=3702 GN=SMC5 PE=2 SV=1
sp|Q12749|SMC6_YEAST	Structural maintenance of chromosomes protein 6 OS=Saccharomyces cerevisiae (strain ATCC 204508 / S288c) OX=559292 GN=SMC6 PE=1 SV=1
sp|Q12188|REC8_YEAST	Meiotic recombination protein REC8 OS=Saccharomyces cerevisiae (strain ATCC 204508 / S288c) OX=559292 GN=REC8 PE=1 SV=1
sp|P23179|SPO11_YEAST	Meiosis-specific protein SPO11 OS=Saccharomyces cerevisiae (strain ATCC 204508 / S288c) OX=559292 GN=SPO11 PE=1 SV=1
sp|P32829|MRE11_YEAST	Double-strand break repair protein MRE11 OS=Saccharomyces cerevisiae (strain ATCC 204508 / S288c) OX=559292 GN=MRE11 PE=1 SV=2
sp|P12753|RAD50_YEAST	DNA repair protein RAD50 OS=Saccharomyces cerevisiae (strain ATCC 204508 / S288c) OX=559292 GN=RAD50 PE=1 SV=1
sp|P25454|RAD51_YEAST	DNA repair protein RAD51 OS=Saccharomyces cerevisiae (strain ATCC 204508 / S288c) OX=559292 GN=RAD51 PE=1 SV=1
sp|Q13315|ATM_HUMAN	Serine-protein kinase ATM OS=Homo sapiens OX=9606 GN=ATM PE=1 SV=4
sp|P38859|DNA2_YEAST	DNA replication ATP-dependent helicase/nuclease DNA2 OS=Saccharomyces cerevisiae (strain ATCC 204508 / S288c) OX=559292 GN=DNA2 PE=1 SV=1
sp|P32841|RE114_YEAST	Meiotic recombination protein REC114 OS=Saccharomyces cerevisiae (strain ATCC 204508 / S288c) OX=559292 GN=REC114 PE=1 SV=3

sp|P53102|MND1_YEAST	Meiotic nuclear division protein 1 OS=Saccharomyces cerevisiae (strain ATCC 204508 / S288c) OX=559292 GN=MND1 PE=1 SV=2
sp|P25453|DMC1_YEAST	Meiotic recombination protein DMC1 OS=Saccharomyces cerevisiae (strain ATCC 204508 / S288c) OX=559292 GN=DMC1 PE=1 SV=1
sp|Q9FKS4|ATR_ARATH	Serine/threonine-protein kinase ATR OS=Arabidopsis thaliana OX=3702 GN=ATR PE=2 SV=2
sp|P48581|RAD17_YEAST	DNA damage checkpoint control protein RAD17 OS=Saccharomyces cerevisiae (strain ATCC 204508 / S288c) OX=559292 GN=RAD17 PE=1 SV=1
sp|P38920|MLH1_YEAST	DNA mismatch repair protein MLH1 OS=Saccharomyces cerevisiae (strain ATCC 204508 / S288c) OX=559292 GN=MLH1 PE=1 SV=2
sp|Q12083|MLH3_YEAST	DNA mismatch repair protein MLH3 OS=Saccharomyces cerevisiae (strain ATCC 204508 / S288c) OX=559292 GN=MLH3 PE=1 SV=1
sp|Q9UQ84|EXO1_HUMAN	Exonuclease 1 OS=Homo sapiens OX=9606 GN=EXO1 PE=1 SV=2
sp|P35187|SGS1_YEAST	ATP-dependent helicase SGS1 OS=Saccharomyces cerevisiae (strain ATCC 204508 / S288c) OX=559292 GN=SGS1 PE=1 SV=1
sp|P38324|SLX1_YEAST	Structure-specific endonuclease subunit SLX1 OS=Saccharomyces cerevisiae (strain ATCC 204508 / S288c) OX=559292 GN=SLX1 PE=1 SV=1
sp|Q12098|SLX4_YEAST	Structure-specific endonuclease subunit SLX4 OS=Saccharomyces cerevisiae (strain ATCC 204508 / S288c) OX=559292 GN=SLX4 PE=1 SV=1
sp|Q04149|MUS81_YEAST	Crossover junction endonuclease MUS81 OS=Saccharomyces cerevisiae (strain ATCC 204508 / S288c) OX=559292 GN=MUS81 PE=1 SV=1
sp|P38257|MMS4_YEAST	Crossover junction endonuclease MMS4 OS=Saccharomyces cerevisiae (strain ATCC 204508 / S288c) OX=559292 GN=MMS4 PE=1 SV=2
sp|P25847|MSH2_YEAST	DNA mismatch repair protein MSH2 OS=Saccharomyces cerevisiae (strain ATCC 204508 / S288c) OX=559292 GN=MSH2 PE=1 SV=2
sp|P25336|MSH3_YEAST	DNA mismatch repair protein MSH3 OS=Saccharomyces cerevisiae (strain ATCC 204508 / S288c) OX=559292 GN=MSH3 PE=1 SV=2
sp|Q03834|MSH6_YEAST	DNA mismatch repair protein MSH6 OS=Saccharomyces cerevisiae (strain ATCC 204508 / S288c) OX=559292 GN=MSH6 PE=1 SV=1
sp|P14242|PMS1_YEAST	DNA mismatch repair protein PMS1 OS=Saccharomyces cerevisiae (strain ATCC 204508 / S288c) OX=559292 GN=PMS1 PE=1 SV=3
sp|P54278|PMS2_HUMAN	Mismatch repair endonuclease PMS2 OS=Homo sapiens OX=9606 GN=PMS2 PE=1 SV=2
HAP2.XP_643321.1|tr|Q75JL6|Q75JL6_DICDI	Hypothetical protein DDB G0276069 [Dictyostelium discoideum AX4]
HAP2.XP_001347424.1	Male gamete fusion factor HAP2, putative [Plasmodium_falciparum_3D7]
HAP2.BAE71144.1	Generative cell specific-1 [Physarum polycephalum]

HOP1.XP_005842819.1	Hypothetical protein CHLNCDRAFT 142584 [Chlorella variabilis]
HOP1.XP_001742099.1|gi|Monbr1|4816|fgenesh1_pg.scaffold_2000132	Hypothetical protein [Monosiga brevicollis MX1]
HOP1.XP_001321336.1	HORMA domain containing protein [Trichomonas vaginalis G3]
PCH2.XP_641871.1|tr|Q54X96|Q54X96_DICDI	Uncharacterized protein AAA ATPase domain-containing protein [Dictyostelium discoideum AX4]
GEX1.XP_637084.1	Hypothetical protein DDB G0287831 [Dictyostelium discoideum AX4]
ZIP4.XP_654536.2	Hypothetical protein EHI 029730 [Entamoeba histolytica HM-1:IMSS]

Supplementary Table S3. KOfam annotation of all sex-associated genes in Symbiodiniaceae. Asterisks (*) indicate significant hits.

Gene	KOfam ID
Fugacium kawagutii	
evm.model.SymbF.scaffold1952.2	K06640
*evm.model.SymbF.scaffold771.2	K10872
*evm.model.SymbF.scaffold1348.3	K10746
*evm.model.SymbF.scaffold345.7	K12854
evm.model.SymbF.scaffold61.7	K08734
*evm.model.SymbF.scaffold1395.4	K10865
evm.model.SymbF.scaffold4961.1	K08737
*evm.model.SymbF.scaffold1411.5	K08991
evm.model.SymbF.scaffold248.30	K10858
*evm.model.SymbF.scaffold1064.19	K10866
*evm.model.SymbF.scaffold3698.1	K15078
*evm.model.SymbF.scaffold1552.2	K06636
evm.model.SymbF.scaffold429.4	K10878
Breviolum minutum	
*B_minutum.evm.model.scaffold3179.1_size61902.2	K06990
*B_minutum.evm.model.scaffold3135.1_size62805.2	K04728
*B_minutum.evm.model.scaffold2481.1_size78112.3	K06642
B_minutum.evm.model.scaffold7157.1_size12992.1	K06640
*B_minutum.evm.model.scaffold524.1_size192337.2	K10872
*B_minutum.evm.model.scaffold1553.1_size112120.8	K10746
*B_minutum.evm.model.scaffold329.1_size228424.10	K15271
*B_minutum.evm.model.scaffold1124.1_size136573.1	K12854
B_minutum.evm.model.scaffold621.1_size179687.2	K18663
*B_minutum.evm.model.scaffold1866.1_size97981.3	K08734
B_minutum.evm.model.scaffold2912.1_size67362.3	K06669
*B_minutum.evm.model.scaffold2842.1_size69055.1	K07238
*B_minutum.evm.model.scaffold2056.1_size90925.4	K10865
*B_minutum.evm.model.scaffold2056.1_size90925.2	K10865
*B_minutum.evm.model.scaffold162.1_size290285.2	K08735
B_minutum.evm.model.scaffold2720.1_size72291.1	K08737
*B_minutum.evm.model.scaffold959.1_size148843.11	K08740
*B_minutum.evm.model.scaffold4251.1_size42412.2	K08741
*B_minutum.evm.model.scaffold4251.1_size42412.2	K08741
B_minutum.evm.model.scaffold2720.1_size72291.1	K08737
*B_minutum.evm.model.scaffold1531.1_size113404.5	K08991
B_minutum.evm.model.scaffold5600.1_size25446.2	K10858

*B_minutum.evm.model.scaffold2483.1_size114807.1	K10866
*B_minutum.evm.model.scaffold3646.1_size68564.1	K10866
*B_minutum.evm.model.scaffold2358.1_size81431.7	K04482
*B_minutum.evm.model.scaffold2553.1_size78617.3	K10730
*B_minutum.evm.model.scaffold113.1_size324549.10	K15078
*B_minutum.evm.model.scaffold346.1_size246720.10	K06636
B_minutum.evm.model.scaffold867.1_size156651.4	K17085
Cladocopium goreaui	
*C_goreaui.evm.model.SymbC1.scaffold1144.2	K04728
C_goreaui.evm.model.SymbC1.scaffold616.12	K06640
C_goreaui.evm.model.SymbC1.scaffold2231.2	K10872
*C_goreaui.evm.model.SymbC1.scaffold3268.6	K10872
*C_goreaui.evm.model.SymbC1.scaffold369.8	K10746
*C_goreaui.evm.model.SymbC1.scaffold6395.1	K06695
C_goreaui.evm.model.SymbC1.scaffold726.9	K15271
*C_goreaui.evm.model.SymbC1.scaffold291.10	K12854
*C_goreaui.evm.model.SymbC1.scaffold1096.5	K08734
C_goreaui.evm.model.SymbC1.scaffold556.4	K01214
*C_goreaui.evm.model.SymbC1.scaffold556.4	K08244
C_goreaui.evm.model.SymbC1.scaffold2722.9	K08739
*C_goreaui.evm.model.SymbC1.scaffold1262.13	K10865
*C_goreaui.evm.model.SymbC1.scaffold11877.1	K08735
*C_goreaui.evm.model.SymbC1.scaffold1556.3	K08741
C_goreaui.evm.model.SymbC1.scaffold2144.10	K08740
*C_goreaui.evm.model.SymbC1.scaffold2144.10	K01493
*C_goreaui.evm.model.SymbC1.scaffold1556.3	K08741
*C_goreaui.evm.model.SymbC1.scaffold123.1	K08991
*C_goreaui.evm.model.SymbC1.scaffold2881.1	K10858
*C_goreaui.evm.model.SymbC1.scaffold8786.2	K10866
C_goreaui.evm.model.SymbC1.scaffold24.270	K03654
*C_goreaui.evm.model.SymbC1.scaffold24.270	K01443
C_goreaui.evm.model.SymbC1.scaffold970.4	K10900
*C_goreaui.evm.model.SymbC1.scaffold879.4	K15078
*C_goreaui.evm.model.SymbC1.scaffold495.10	K06636
C_goreaui.evm.model.SymbC1.scaffold1772.3	K06636
C_goreaui.evm.model.SymbC1.scaffold13117.2	K10878
***Cladocopium* sp. C92**	
*Cladocopium.evm.model.scaffold460.1.12	K06642
Cladocopium.evm.model.scaffold137.1.21	K06640
*Cladocopium.evm.model.scaffold78.1.21	K10872
*Cladocopium.evm.model.scaffold1669.1.4	K10872
*Cladocopium.evm.model.scaffold2340.1.4	K10746

*Cladocopium.evm.model.scaffold1557.1.3	K06695
*Cladocopium.evm.model.scaffold24.1.22	K15271
*Cladocopium.evm.model.scaffold679.1.10	K12854
Cladocopium.evm.model.scaffold1966.1.3	K18663
*Cladocopium.evm.model.scaffold823.1.4	K08734
Cladocopium.evm.model.scaffold1562.1.10	K09542
Cladocopium.evm.model.scaffold94.1.35	K08739
*Cladocopium.evm.model.scaffold5666.1.1	K08735
*Cladocopium.evm.model.scaffold1694.1.9	K08741
*Cladocopium.evm.model.scaffold1470.1.1	K08737
*Cladocopium.evm.model.scaffold1694.1.9	K08741
*Cladocopium.evm.model.scaffold1470.1.1	K08737
Cladocopium.evm.model.scaffold673.1.20	K08991
*Cladocopium.evm.model.scaffold740.1.1	K10858
Cladocopium.evm.model.scaffold880.1.21	K10872
Cladocopium.evm.model.scaffold880.1.18	K04482
Cladocopium.evm.model.scaffold42.1.3	K10900
*Cladocopium.evm.model.scaffold593.1.5	K15078
*Cladocopium.evm.model.scaffold2909.1.3	K07374
*Cladocopium.evm.model.scaffold490.1.4	K06636
Cladocopium.evm.model.scaffold3311.1.2	K06636
Cladocopium.evm.model.scaffold14.1.27	K10878
Symbiodinium microadriaticum	
*S_microadriaticum.evm.model.Smic.scaffold222_size825431.15	K04728
S_microadriaticum.evm.model.Smic.scaffold119_size1050411.34	K06640
*S_microadriaticum.evm.model.Smic.scaffold678_size401019.26	K10872
*S_microadriaticum.evm.model.Smic.scaffold326_size661508.1	K10746
*S_microadriaticum.evm.model.Smic.scaffold188_size896232.23	K10746
*S_microadriaticum.evm.model.Smic.scaffold1550_size119722.1	K06695
S_microadriaticum.evm.model.Smic.scaffold733_size375764.2	K15271
S_microadriaticum.evm.model.Smic.scaffold1369_size161076.3	K12854
*S_microadriaticum.evm.model.Smic.scaffold1323_size174227.5	K08734
S_microadriaticum.evm.model.Smic.scaffold90_size1176205.61	K12819
S_microadriaticum.evm.model.Smic.scaffold53_size1398322.44	K10865
*S_microadriaticum.evm.model.Smic.scaffold1099_size246254.12	K10865
*S_microadriaticum.evm.model.Smic.scaffold1313_size176020.10	K08737
*S_microadriaticum.evm.model.Smic.scaffold1313_size176020.10	K08737
*S_microadriaticum.evm.model.Smic.scaffold446_size553009.8	K10866
S_microadriaticum.evm.model.Smic.scaffold464_size542056.13	K04482
*S_microadriaticum.evm.model.Smic.scaffold165_size944511.67	K10730
*S_microadriaticum.evm.model.Smic.scaffold394_size591120.1	K15078
*S_microadriaticum.evm.model.Smic.scaffold251_size768168.49	K06669

*S_microadriaticum.evm.model.Smic.scaffold829_size332361.28	K17085
***Cladocopium* sp.C15 MMETSP1370**	
*Symbiodinium-sp-C15-MMETSP1370--20130923_4779_1_1	K10872
*Symbiodinium-sp-C15-MMETSP1370--20130923_6475_1_1	K12854
*Symbiodinium-sp-C15-MMETSP1370--20130923_47373_1_1	K04482
***Cladocopium* sp.C1 MMETSP1367**	
Symbiodinium-sp-C1-MMETSP1367--20140214_8898_1_1	K12261
*Symbiodinium-sp-C1-MMETSP1367--20140214_21889_1_1	K12854
*Symbiodinium-sp-C1-MMETSP1367--20140214_12426_1_1	K10730
*Symbiodinium-sp-C1-MMETSP1367--20140214_9361_1_1	K15078
*Symbiodinium-sp-C1-MMETSP1367--20140214_3193_1_1	K06636
**Durusdinium_sp.D1a_MMETSP1377**	
*Ssp_D1a_MMETSP1377-20130617_74152_1_1	K10872
Ssp_D1a_MMETSP1377-20130617_57944_1_1	K18719
Ssp_D1a_MMETSP1377-20130617_73383_1_1	K04482
Symbiodinium tridacnidorum	
S_tridacnidorum.evm.model.scaffold250.1.5	K06640
*S_tridacnidorum.evm.model.scaffold36.1.4	K10746
*S_tridacnidorum.evm.model.scaffold2771.1.2	K10746
*S_tridacnidorum.evm.model.scaffold4406.1.1	K10746
*S_tridacnidorum.evm.model.scaffold3290.1.2	K12854
*S_tridacnidorum.evm.model.scaffold5751.1.1	K08734
S_tridacnidorum.evm.model.scaffold6321.1.2	K09855
S_tridacnidorum.evm.model.scaffold3079.1.7	K23285
S_tridacnidorum.evm.model.scaffold842.1.4	K10865
*S_tridacnidorum.evm.model.scaffold4395.1.1	K08740
*S_tridacnidorum.evm.model.scaffold1895.1.2	K08741
*S_tridacnidorum.evm.model.scaffold1895.1.2	K08741
S_tridacnidorum.evm.model.scaffold5550.1.3	K08737
*S_tridacnidorum.evm.model.scaffold1250.1.7	K10866
S_tridacnidorum.evm.model.scaffold4739.1.1	K04482
*S_tridacnidorum.evm.model.scaffold2023.1.2	K15078
*S_tridacnidorum.evm.model.scaffold1600.1.4	K06669
**Symbiodinium tridacnidorum (hybrid genome)**	
*Stri_CCMP2592.gene63.mRNA1:MRE11	K10865
Stri_CCMP2592.gene2631.mRNA1:SLX1	K15271
*Stri_CCMP2592.gene5864.mRNA1:MND1	K13525
*Stri_CCMP2592.gene15583.mRNA1:SMC3	K06669
*Stri_CCMP2592.gene17916.mRNA1:ATM	K04728
Stri_CCMP2592.gene17916.mRNA1:ATM	K02543
*Stri_CCMP2592.gene19540.mRNA1:DNA2	K14326
*Stri_CCMP2592.gene21650.mRNA1:RAD51	K04482

*Stri_CCMP2592.gene23678.mRNA1:MSH6	K08737
Stri_CCMP2592.gene23678.mRNA1:MSH6	K08735
*Stri_CCMP2592.gene23827.mRNA1:RAD50	K10866
Stri_CCMP2592.gene23827.mRNA1:RAD50	K16808
Stri_CCMP2592.gene24609.mRNA1:SGS1	K10901
Stri_CCMP2592.gene26081.mRNA1:ATR	K06640
*Stri_CCMP2592.gene30157.mRNA1:DMC1	K03553
*Stri_CCMP2592.gene30529.mRNA1:SMC1	K06636
*Stri_CCMP2592.gene30726.mRNA1:PMS2	K10858
*Stri_CCMP2592.gene31829.mRNA1:MLH1	K08734
*Stri_CCMP2592.gene33878.mRNA1:MSH4	K08740
*Stri_CCMP2592.gene34122.mRNA1:EXO1	K10746
*Stri_CCMP2592.gene37007.mRNA1:MSH5	K08741
*Stri_CCMP2592.gene37221.mRNA1:MER3	K12854
Stri_CCMP2592.gene38648.mRNA1:MLH3	K08739
***Breviolum minutum* SSB01**	
*Symbiodinium_B_SSB01.transcriptome.s6_4895_m.13582_1	K04728
Symbiodinium_B_SSB01.transcriptome.s6_5377_m.14886_1	K06640
*Symbiodinium_B_SSB01.transcriptome.s6_14533_m.38129_1	K10872
*Symbiodinium_B_SSB01.transcriptome.s6_30035_m.67239_1	K08734
Symbiodinium_B_SSB01.transcriptome.s6_13435_m.35571_1	K09855
Symbiodinium_B_SSB01.transcriptome.s6_32263_m.69959_1	NA
Symbiodinium_B_SSB01.transcriptome.s6_43512_m.84223_1	K12472
*Symbiodinium_B_SSB01.transcriptome.s6_4388_m.12243_1	K10858
*Symbiodinium_B_SSB01.transcriptome.s6_8603_m.23390_1	K10866
*Symbiodinium_B_SSB01.transcriptome.s6_34754_m.73761_1	K04482
*Symbiodinium_B_SSB01.transcriptome.s6_2449_m.6900_1	K06669
Symbiodinium_B_SSB01.transcriptome.s6_4724_m.13143_1	K10878
***Cladocopium goreaui* MI SCF055**	
C1_MI.TR47116_c0_g1_i1|m.78967	K10878
*C1_MI.TR34615_c0_g1_i1|m.50332	K08741
Symbiodinium_C1_MI_SCF055.transcriptome.TR57407_c0_g1_i1_m.117089_1	K06642
*Symbiodinium_C1_MI_SCF055.transcriptome.TR48870_c0_g2_i1_m.84545_1	K10872
Symbiodinium_C1_MI_SCF055.transcriptome.TR48870_c0_g2_i2_m.84547_1	K10872
*Symbiodinium_C1_MI_SCF055.transcriptome.TR57112_c0_g1_i1_m.115733_1	K18663
Symbiodinium_C1_MI_SCF055.transcriptome.TR44313_c0_g1_i1_m.71806_1	K12472
*Symbiodinium_C1_MI_SCF055.transcriptome.TR48734_c0_g1_i1_m.84110_1	K10730
*Symbiodinium_C1_MI_SCF055.transcriptome.TR47318_c0_g1_i1_m.79572_1	K06669
***Cladocopium goreaui* WSY**	
Symbiodinium_C1_WSY.transcriptome.TR60325_c0_g3_i1_m.96902_1	K06695
Symbiodinium_C1_WSY.transcriptome.TR113068_c0_g1_i1_m.194897_1	K17945
C1_WSY.TR26080_c0_g1_i1|m.29390	K08740

*C1_WSY.TR15578_c0_g1_i1|m.18543	K08741
C1_WSY.TR41849_c0_g2_i1|m.54442	K10878
Symbiodinium_C1_WSY.transcriptome.TR60338_c0_g1_i1_m.96919_1	K12261
Symbiodinium_C1_WSY.transcriptome.TR76569_c0_g1_i1_m.172282_1	K09855
*Symbiodinium_C1_WSY.transcriptome.TR61627_c0_g1_i1_m.100905_1	K10730
*Symbiodinium_C1_WSY.transcriptome.TR43103_c0_g1_i1_m.56284_1	K15078
*Symbiodinium_C1_WSY.transcriptome.TR68277_c0_g1_i1_m.124779_1	K06669
***Cladocopium* sp. Davies**	
Symbiodinium_C_Davies.transcriptome.comp225360_c0_seq1_m.100867_1	K06642
Symbiodinium_C_Davies.transcriptome.comp224937_c0_seq1_m.100350_1	K06640
*Symbiodinium_C_Davies.transcriptome.comp164938_c0_seq1_m.55930_1	K10872
*Symbiodinium_C_Davies.transcriptome.comp206048_c0_seq1_m.82662_1	K18663
*Symbiodinium_C_Davies.transcriptome.comp45170_c0_seq1_m.9064_1	K08734
Symbiodinium_C_Davies.transcriptome.comp43714_c0_seq1_m.8199_1	K06669
Symbiodinium_C_Davies.transcriptome.comp85553_c0_seq1_m.23626_1	K19657
Symbiodinium_C_Davies.transcriptome.comp196372_c0_seq1_m.75326_1	K12472
*Symbiodinium_C_Davies.transcriptome.comp114774_c0_seq1_m.35894_1	K08991
*Symbiodinium_C_Davies.transcriptome.comp117548_c0_seq1_m.41487_1	K10858
*Symbiodinium_C_Davies.transcriptome.comp215158_c0_seq1_m.90798_1	K10730
*Symbiodinium_C_Davies.transcriptome.comp113365_c0_seq1_m.32972_1	K15078
*Symbiodinium_C_Davies.transcriptome.comp113365_c0_seq2_m.32973_1	K15078
*Symbiodinium_C_Davies.transcriptome.comp214734_c0_seq3_m.90289_1	K06636
Symbiodinium_C_Davies.transcriptome.comp67549_c0_seq1_m.18664_1	K10878
***Symbiodinium microadriaticum* CassKB8**	
Symbiodinium_CladeA_CassKB8.transcriptome.kb8_rep_c6053_m.17231_1	K04986
*Symbiodinium_CladeA_CassKB8.transcriptome.kb8_rep_c3666_m.10303_1	K10872
*Symbiodinium_CladeA_CassKB8.transcriptome.kb8_c7486_m.21514_1	K10746
Symbiodinium_CladeA_CassKB8.transcriptome.kb8_rep_c2922_m.8218_1	K12854
Symbiodinium_CladeA_CassKB8.transcriptome.kb8_c4351_m.12261_1	K21348
*Symbiodinium_CladeA_CassKB8.transcriptome.kb8_rep_c5255_m.14910_1	K10866
*Symbiodinium_CladeA_CassKB8.transcriptome.kb8_rep_c1564_m.4374_1	K04482
Symbiodinium_CladeA_CassKB8.transcriptome.kb8_c12156_m.35541_1	K10730
*Symbiodinium_CladeA_CassKB8.transcriptome.kb8_c5225_m.14814_1	K06669
***Breviolum minutum* Mf1.05b**	
Symbiodinium_CladeB1_Mf1_05b.transcriptome.mf105_rep_c4226_m.8581_1	K04728
Symbiodinium_CladeB1_Mf1_05b.transcriptome.mf105_rep_c2084_m.4214_1	K19801
Symbiodinium_CladeB1_Mf1_05b.transcriptome.mf105_rep_c654_m.1287_1	K10746
Symbiodinium_CladeB1_Mf1_05b.transcriptome.mf105_rep_c6015_m.12101_1	K10878
Breviolum aenigmaticum	
Symbiodinium_aenigmaticum.transcriptome.comp7900_c0_seq1_m.12231_1	K09855
Symbiodinium_aenigmaticum.transcriptome.comp15743_c0_seq1_m.24273_1	K17589

Symbiodinium_aenigmaticum.transcriptome.comp15219_c0_seq1_m.23381_1	K22804
*Symbiodinium_aenigmaticum.transcriptome.comp15523_c0_seq1_m.23904_1	K08737
*Symbiodinium_aenigmaticum.transcriptome.comp15523_c0_seq1_m.23904_1	K08737
*Symbiodinium_aenigmaticum.transcriptome.comp17131_c0_seq1_m.26541_1	K04482
*Symbiodinium_aenigmaticum.transcriptome.comp12038_c0_seq1_m.18264_1	K06669
*Symbiodinium_aenigmaticum.transcriptome.comp11005_c0_seq1_m.16705_1	K06669
Breviolum pseudominutum	
Symbiodinium_pseudominutum.transcriptome.comp6106_c0_seq1_m.8268_1	K06640
*Symbiodinium_pseudominutum.transcriptome.comp19625_c0_seq1_m.26136_1	K10872
*Symbiodinium_pseudominutum.transcriptome.comp22792_c0_seq1_m.30598_1	K06695
Symbiodinium_pseudominutum.transcriptome.comp12447_c0_seq1_m.17418_1	K09855
*Symbiodinium_pseudominutum.transcriptome.comp25234_c0_seq1_m.33962_1	K10858
*Symbiodinium_pseudominutum.transcriptome.comp21438_c0_seq1_m.28586_1	K06669
Symbiodinium_pseudominutum.transcriptome.comp28655_c0_seq1_m.38901_1	K10878
Breviolum psygmophilum	
Symbiodinium_psygophilum.transcriptome.comp35370_c0_seq2_m.51960_1	K06640
*Symbiodinium_psygophilum.transcriptome.comp25554_c0_seq1_m.30375_1	K10872
Symbiodinium_psygophilum.transcriptome.comp42357_c0_seq1_m.62735_1	K17589
Symbiodinium_psygophilum.transcriptome.comp11830_c0_seq1_m.11064_1	K08739
Symbiodinium_psygophilum.transcriptome.comp40604_c0_seq1_m.61099_1	K22804
Symbiodinium_psygophilum.transcriptome.comp6497_c0_seq1_m.6195_1	K09855
*Symbiodinium_psygophilum.transcriptome.comp44385_c0_seq1_m.64835_1	K04482
*Symbiodinium_psygophilum.transcriptome.comp39462_c0_seq1_m.60012_1	K06669
*Symbiodinium_psygophilum.transcriptome.comp13404_c0_seq1_m.12941_1	K06669
Symbiodinium_psygophilum.transcriptome.comp12790_c0_seq1_m.12231_1	K10878
Effrenium voratum	
*Ssp_CCMP421_MMETSP1110-20121109_77407_1_1	K10746
*Ssp_CCMP421_MMETSP1110-20121109_13169_1_1	K12854
*Ssp_CCMP421_MMETSP1110-20121109_7895_1_1	K18663
*Ssp_CCMP421_MMETSP1110-20121109_5706_1_1	K10865
*Ssp_CCMP421_MMETSP1110-20121109_39034_1_1	K08735
*Ssp_CCMP421_MMETSP1110-20121109_64844_1_1	K08991
Ssp_CCMP421_MMETSP1110-20121109_18304_1_1	K10858
*Ssp_CCMP421_MMETSP1110-20121109_77145_1_1	K10858
*Ssp_CCMP421_MMETSP1110-20121109_6576_1_1	K10866
*Ssp_CCMP421_MMETSP1110-20121109_35057_1_1	K04482
*Ssp_CCMP421_MMETSP1110-20121109_69154_1_1	K04482

Ssp_CCMP421_MMETSP1110-20121109_8409_1_1	K10900
*Ssp_CCMP421_MMETSP1110-20121109_33380_1_1	K15078
*Ssp_CCMP421_MMETSP1110-20121109_39870_1_1	K06636
Ssp_CCMP421_MMETSP1110-20121109_73845_1_1	K06636
*Ssp_CCMP421_MMETSP1110-20121109_55608_1_1	K06636
*Ssp_CCMP421_MMETSP1110-20121109_21182_1_1	K06669
*Ssp_CCMP421_MMETSP1110-20121109_15219_1_1	K06669
Symbiodinium natans	
ATR.S_natans.evm.model.scaffold_129.5	K06640
*EXO1.S_natans.evm.model.scf7180000015630.22	K10746
*EXO1.S_natans.evm.model.scaffold_185.15	K10746
*HOP2.S_natans.evm.model.scaffold_83.16	K06695
*MER3.S_natans.evm.model.scaffold_540.24	K12854
MER3.S_natans.evm.model.scaffold_210.45	K18663
*MLH1.S_natans.evm.model.scaffold_548.11	K08734
*MRE11.S_natans.evm.model.scaffold_148.47	K10865
*MSH4.S_natans.evm.model.scaffold_337.43	K08741
*MSH4.S_natans.evm.model.scf7180000015290.14	K08737
*MSH5.S_natans.evm.model.scaffold_337.43	K08741
*MSH6.S_natans.evm.model.scf7180000015290.14	K08737
PMS2.S_natans.evm.model.scaffold_423.26	K10858
*RAD51.S_natans.evm.model.scaffold_86.17	K04482
*SGS1.new.trimal.S_natans.evm.model.scaffold_532.32	K10730
*SLX1.S_natans.evm.model.scaffold_116.47	K15078
SMC1.S_natans.evm.model.scaffold_10.1	K06636
SMC1.S_natans.evm.model.scaffold_10.44	K06636
*SMC1.S_natans.evm.model.scf7180000015605.9	K06636
*SMC3.S_natans.evm.model.scf7180000014684.13	K06669
*SPO11.S_natans.evm.model.scaffold_704.17	K17085
